# Synthesis, and antibacterial activities of novel 1,3,4a,9-tetraza-4*H*-fluoren-2-amines incorporating phenoxy-*N*-arylacetamide, pyrazole, and 2-(4-(1-phenyl-1*H*-pyrazol-3-yl)phenoxy)-*N*-arylacetamide moieties

**DOI:** 10.1186/s13065-025-01421-5

**Published:** 2025-03-08

**Authors:** Reham E. Abdelwahab, Ahmed H. M. Elwahy, Nada S. Ibrahim, Amr M. Abdelmoniem, Ismail A. Abdelhamid

**Affiliations:** 1 Department of Chemistry, Faculty of Science, Cairo University, Giza, 12613 Egypt; 2 Department of Chemistry (Biochemistry Division), Faculty of Science, Cairo University, Giza, 12613 Egypt

**Keywords:** Ring annelation, 1-(1*H*-benzo[*d*]imidazol-2-yl)guanidine, 1,3,4a,9-tetraza-4*H*-fluoren-2-amines, phenoxy-*N*-arylacetamide, *Trans* esterification, Antibacterial activity

## Abstract

A ring annelation reaction was used to successfully prepare benzo[4,5]imidazo[1,2-*a*][1,3,5]triazines (Systematic Name: 1,3,4a,9-tetraza-4*H*-fluoren-2-amines) tethered to phenoxy-*N*-arylacetamide, pyrazole, and 2-(4-(1-phenyl-1*H*-pyrazol-3-yl)phenoxy)-*N*-arylacetamide moieties utilizing 1-(1*H*-benzo[*d*]imidazol-2-yl)guanidine and the proper aldehydes as precursors. 2-(Phenylamino)ethyl fragment of compound **7** was cleaved off and compound **8** was formed. The constitutions of the novel compounds were confirmed based on spectral data. The antibacterial activity was evaluated for the prepared compounds against two gram-negative and two gram-positive bacteria. Among them, compound **12b** (inhibition zone 16 ± 0.7 mm) was the most promising against *S. aureus* compared to Gentamycin (15 ± 0 mm). Also, compounds **5a** and **5d** exerted comparable antibacterial activity (inhibition zones 13 ± 1.4 and 13 ± 2.1 mm), respectively to Gentamycin against *S. aureus*. Minimum inhibitory concentration (MIC) evaluation against *S. aureus* showed that compound **12b** had the lowest MIC value (78.1 µg/mL).

**Supplementary Information:**

The online version contains supplementary material available at 10.1186/s13065-025-01421-5.

## Introduction

**Fig. 1:**
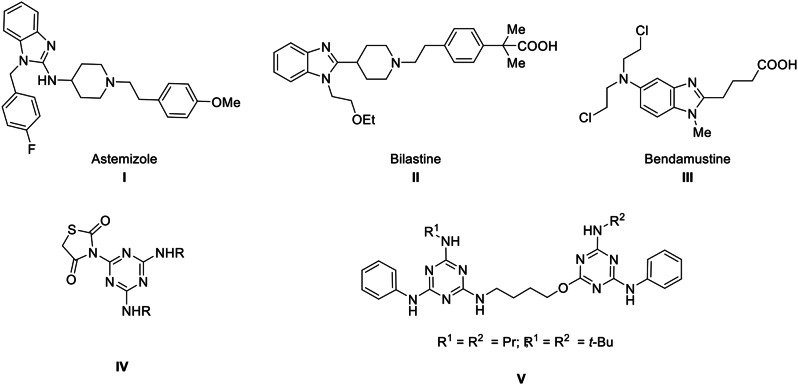
Some drugs containing benzimidazole or [1,3,5]triazine cores

Benzimidazoles are the fundamental component of a wide range of biochemical and medicinal substances with varying chemical and pharmacological properties. Numerous derivatives of benzimidazoles have a variety of biological characteristics, including antitumor [1–6], antifungal [7, 8], antiviral [9–11], antihistaminic [12–14] antibacterial [15, 16], and anticonvulsant activity [17, 18]. Representative examples of drugs containing benzimidazole moiety are depicted in Fig. 1 (compounds **I** [19–21], **II** [22–24], **III** [25–27]).

Moreover, triazines are benzene-like six-membered planar structures having three nitrogen atoms [28]. The triazine scaffold is the most well-known heterocycle, with a wide spectrum of biological activity [28–31]. There are three triazine isomers, namely, 1,2,4-triazine, 1,2,3-triazine, and 1,3,5-triazine. 1,3,5-Triazine is the most studied due to its distinct chemical structure and medicinal capabilities [30, 31]. For example, 1,3,5-triazine-thiazolidine-dione **IV** [30] has been reported as a DPP-4 inhibitor with antibacterial activity targeting the S1 pocket for the treatment of type 2 diabetes [30] (Fig. 1). Also, triazine dimer **V** [31] has been reported as an antileishmanial agent (Fig. 1) [31].

Besides, it was noted that compounds with acetamide linkages as core structures have drawn a lot of interest because of their possible therapeutic applications, including anticancer [32, 33], haemolytic [34], antioxidant [35], antitubercular [36], antiurease [34], antimicrobial [34, 37, 38], anti-inflammatory [39], anticonvulsant [40], analgesic [38, 39], anti-COVID-19 [41], and antituberculosis [42].

Moreover, pyrazoles, a five-membered heterocycle with two neighboring nitrogen atoms, are the fundamental structures found in a variety of compounds with diverse biological activities such as anticancer [43, 44], anti-inflammatory [45, 46], antimicrobial [46, 47], antioxidant [48, 49], and anticonvulsant [50] activities. Furthermore, bis-heterocycles, which consist of two bioactive heterocycles linked by a flexible linker, have been reported to possess plant growth regulative, anticancer, antibacterial, and fungicidal properties [51–55]. They can also be used as chelating agents, electrical conducting compounds [56], and metal ligands [57].

Fluorene is one example of a polyaromatic hydrocarbon (PAH), an essential precursor used in manufacturing as a component of plastics, insecticides, resins, dyes, and medications [58–60]. It was found that replacing one or more carbon atoms with heteroatoms results in significant alterations of its biological activity [61, 62]. Thus, in continuation of our interest in the synthesis of bioactive heterocycles [63–87], we aim to prepare hybrid heterocycles based on 1,3,4a,9-tetraza-4*H*-fluoren-2-amines tethered phenoxy-*N*-arylacetamide, and pyrazole moiety.

## Results and discussion

**Scheme 1:**
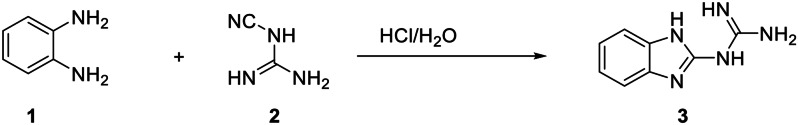
Synthesis of 1-(1*H*-benzo[*d*]imidazol-2-yl)guanidine **3**

According to the preceding general procedure, the reaction of *o*-phenylenediamine **1** with cyanoguanidine **2** in a refluxing water-HCl mixture produces 1-(1*H*-benzo[*d*]imidazol-2-yl)guanidine **3** (Scheme 1).

**Scheme 2:**
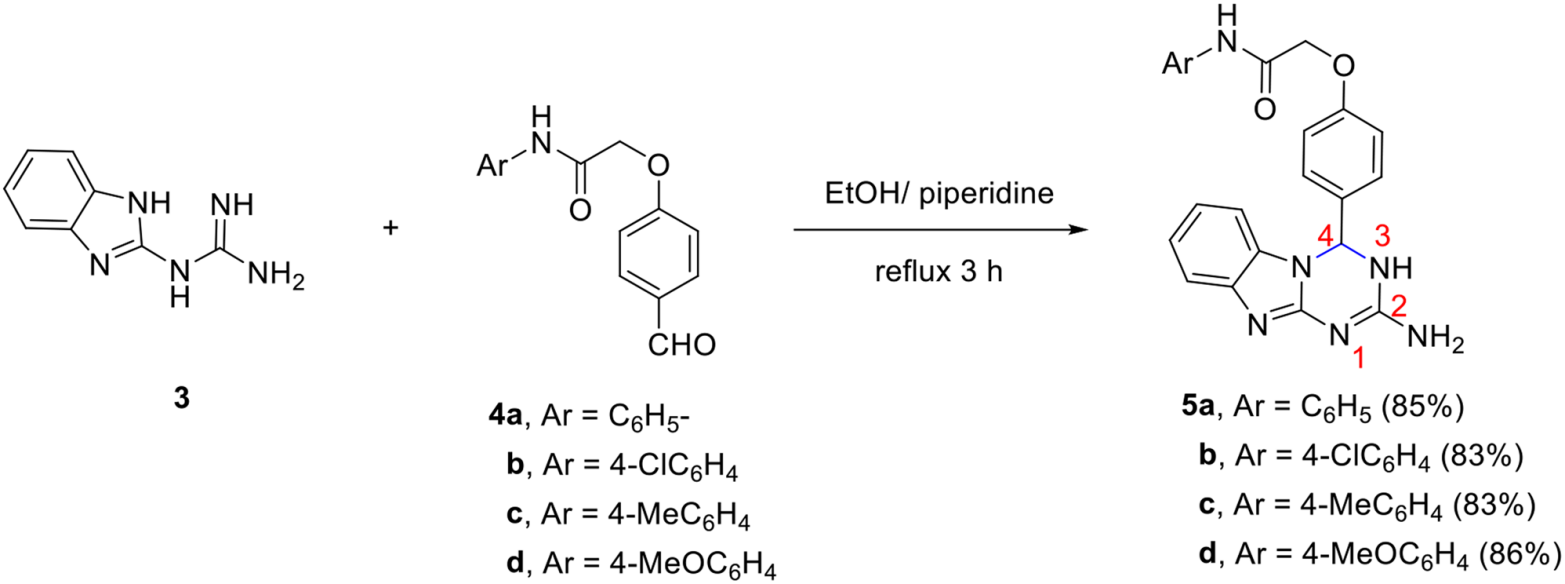
Synthesis of 2-(4-(dihydrobenzo[4,5]imidazo[1,2-*a*][1,3,5]triazin-4-yl)phenoxy)-*N*-arylacetamides **5a-d**

The reaction of 1-(1*H*-benzo[*d*]imidazol-2-yl)guanidine **3** with 2-(4-formylphenoxy)-*N*-arylacetamides **4a-d** in ethanol in the presence of piperidine as a basic catalyst leads to the formation of 2-(4-(2-amino-3,4-dihydrobenzo[4,5]imidazo[1,2-*a*][1,3,5]triazin-4-yl)phenoxy)-*N*-phenylacetamides **5a-d** in which the benzo[4,5]imidazo[1,2-*a*][1,3,5]triazine moiety is linked to phenoxy-*N*-arylacetamide moieties (Scheme 2).

The constitution of the formed products was proved based on spectral data. For example, the mass spectrum of **5a** revealed a molecular ion peak at m/z 412. The IR spectrum of compound **5a** showed characteristic N-H stretching bands at $\:\stackrel{-}{\nu\:\:}$3449 and 3325 cm^− 1^ and a strong absorption band at $\:\stackrel{-}{\nu\:}\:$1674 cm^− 1^ for the carbonyl group. The ^1^H NMR spectrum displayed a singlet at δ 4.69 ppm for OCH_2_ protons besides a multiplet at δ 6.68–7.62 ppm integrated for 14 protons corresponding to the aromatic protons and *H*-4. It also demonstrated three singlet signals (exchangeable with D_2_O) at δ 6.39 (2 H), 7.98 (1H), and 10.06 (1H) ppm assigned to NH_2_ and 2 NH protons. In addition, the ^13^C NMR showed peaks at δ 65.6 and 67.1 ppm for aliphatic carbons O*C*H_2_ and *C*-4 as well as a characteristic peak at δ 166.4 for the amide group. Peaks of the aromatic carbons appear at their appropriate position.

**Fig. 2:**
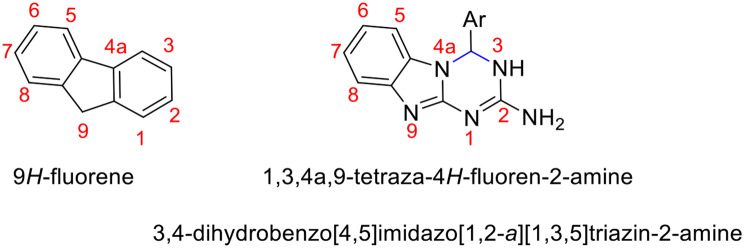
Structures of 9*H*-fluorene and1,3,4a,9-tetraza-4*H*-fluoren-2-amines **5**

Compounds **5** can be seen as 1,3,4a,9-tetraza-4*H*-fluoren-2-amines, as illustrated in Fig. 2. It has been observed that replacing one or more carbon atoms of a carbocyclic molecule with heteroatoms results in significant alterations of its biological activity [88–91].

**Fig. 3:**
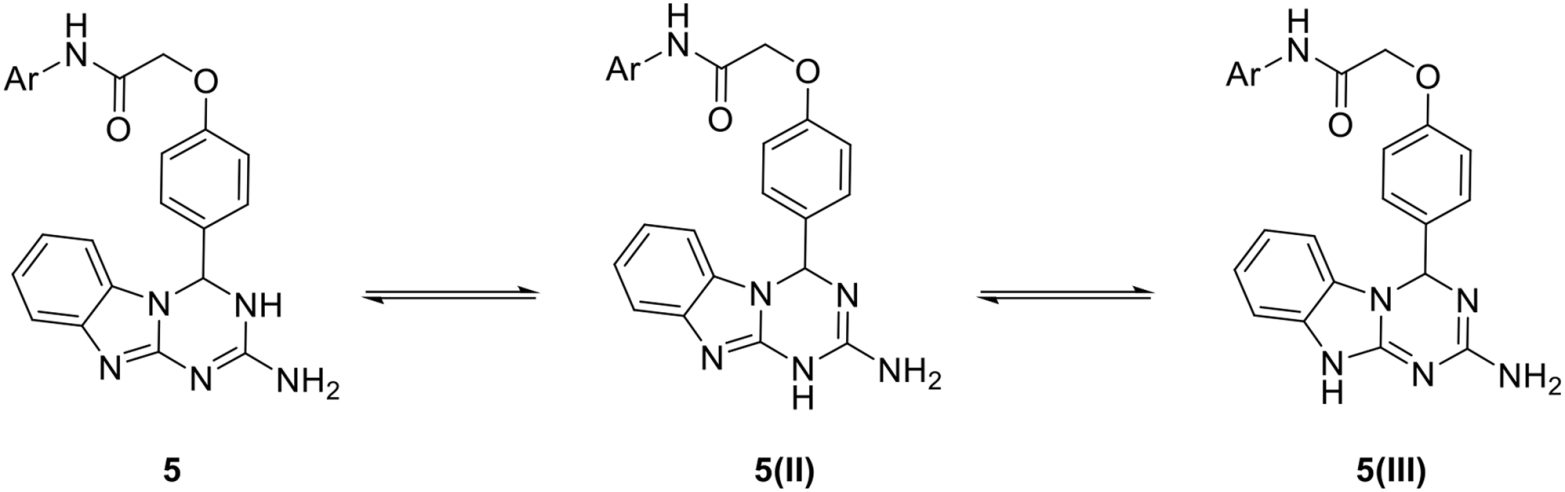
Isomeric structures of [4,5]imidazo[1,2-*a*][1,3,5]triazines **5**

However, the formed dihydrobenzo[4,5]imidazo[1,2-*a*][1,3,5]triazine could exist in three tautomeric structures, namely 3,4-dihydro- **5**, 1,4-dihydro- **5(I)**, and 4,10-dihydro- **5(II)** tautomeric forms, the 3,4-dihydro- isomer is predominant in the crystal form, as indicated in related work [92] (Fig. 3).

**Scheme 3:**
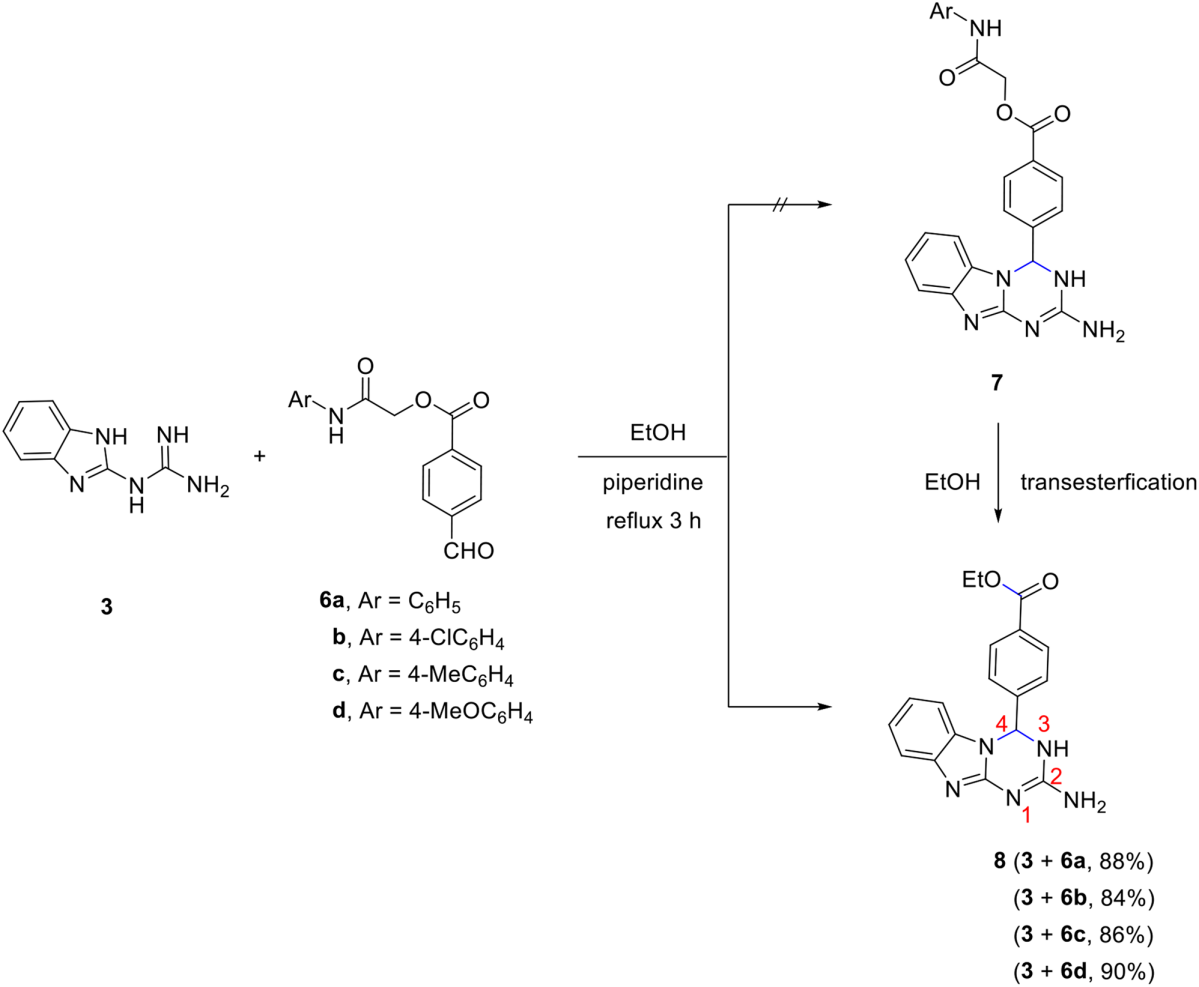
Synthesis of ethyl 4-(2-amino-3,4-dihydrobenzo[4,5]imidazo[1,2-*a*][1,3,5]triazin-4-yl)benzoate **8**

On the other hand, trials to prepare the benzo[4,5]imidazo[1,2-*a*][1,3,5]triazines **7** which are linked to benzoyloxyacetamide *via* the direct reaction of **3** with 2-oxo-2-(phenylamino)ethyl 4-formylbenzoate derivatives **6a-d** under the same condition of absolute ethanol at reflux in presence of a catalytic amount of piperidine did not succeed. Instead, in all these examples, only ethyl 4-(2-amino-3,4-dihydrobenzo[4,5]imidazo[1,2-*a*][1,3,5]triazin-4-yl)benzoate **8** was obtained as the sole product (Scheme 3).

**Scheme 4:**
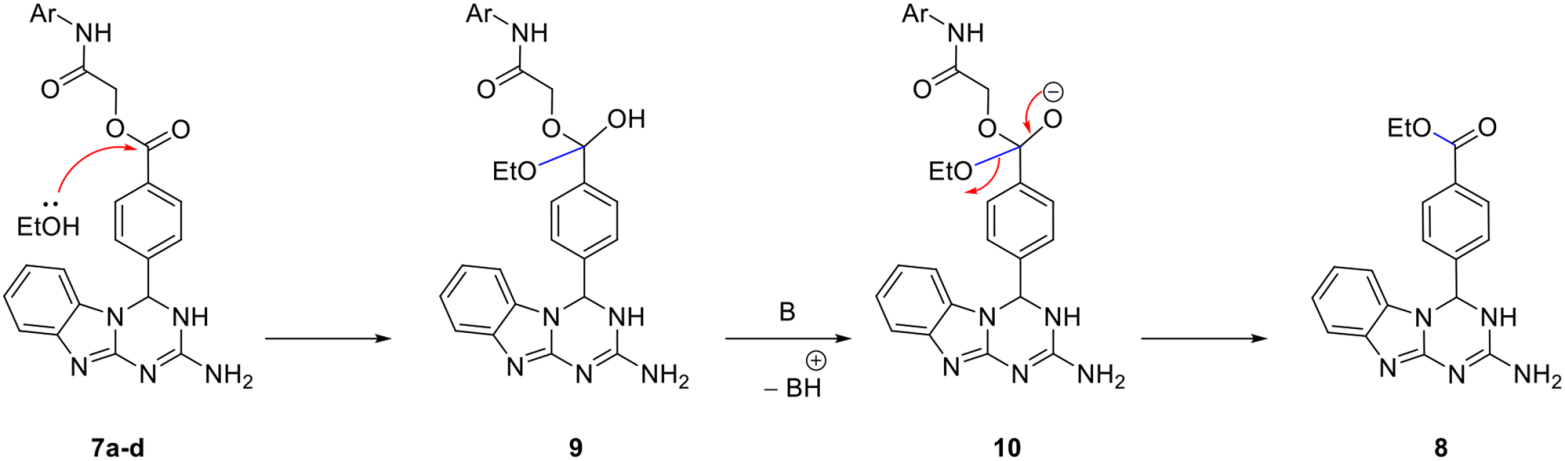
Unexpected formation of ethyl 4-(2-amino-3,4-dihydrobenzo[4,5]imidazo[1,2-*a*][1,3,5]triazin-4-yl)benzoate **8**

Compound **8** was formed due to the transesterification of the initially formed **7a-d** as indicated in the following mechanism. The nucleophilic attack of ethanol to the carbonyl ester of **7** leads to the tetrahedral intermediate **9** that was deprotonated in the presence of a basic catalyst (piperidine) into the intermediate **10** that affords the final isolable product **8** (Scheme 4).

The chemical constitution of compound **8** was undoubtedly confirmed by the aid of spectral tools and elemental analysis. The mass spectrum showed a molecular ion peak at *m/z* 335, consistent with the proposed structure. The IR spectrum revealed the presence of a characteristic absorption band at $\:\stackrel{-}{\nu\:}$ 1713 cm^− 1^ for the ester carbonyl stretch, and broad absorption bands at $\:\stackrel{-}{\nu\:}$ 3418 and 3248 cm^− 1^ for NH_2_ and NH group. The ^1^H NMR further confirmed the transesterification as it showed the absence of any peaks for OC*H*_2_ and amide N*H* protons. Instead, ethoxy protons appeared as a characteristic triplet and quartet at δ 1.26 and 4.25 ppm (p*J* = 7.2 Hz). NH_2_ and NH protons gave peaks at δ 6.42 (2 H) and 8.09 (1H) ppm, respectively. Other peaks for aromatic protons and H-4 exist in their expected position. ^13^C NMR spectrum showed peaks for carbons of ethoxy group at δ 14.2 and 61.1 ppm. It revealed also peaks at δ 65.4 ppm for C-4, and at δ 165.3 ppm for ester carbonyl. Peaks of other aromatic carbons appeared as expected.

**Scheme 5:**
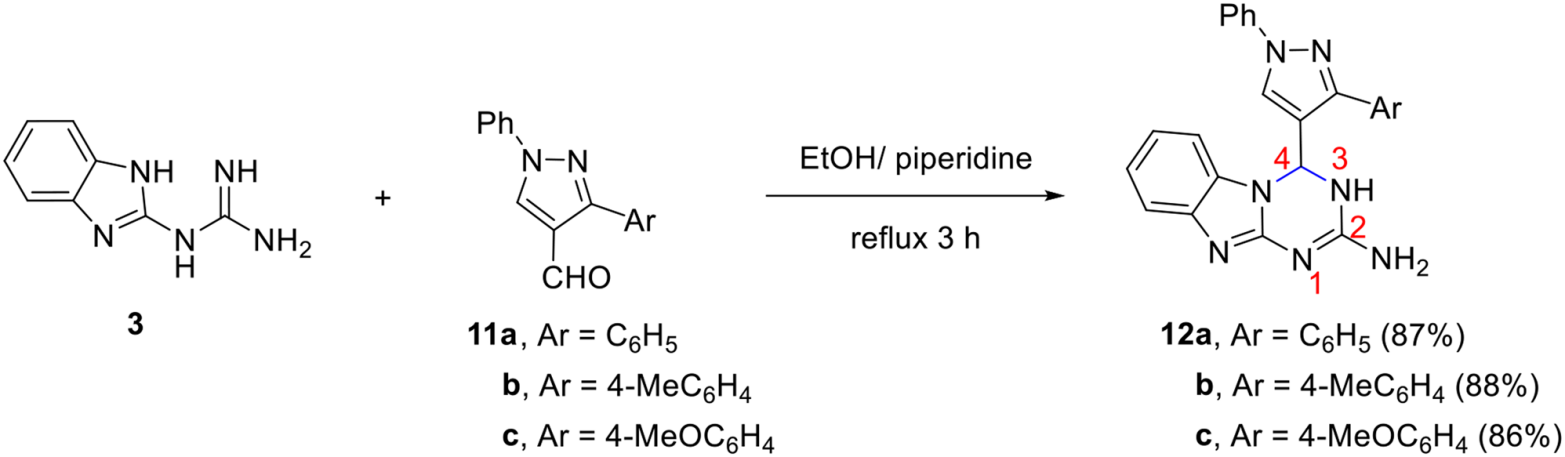
Synthesis of pyrazole-containing 1,3,4a,9-tetraza-4*H*-fluoren-2-amines **12a-c**

To achieve the concept of molecular hybridization, we attempt to introduce the biologically active pyrazole ring into the structure of 1,3,4a,9-tetraza-4*H*-fluoren-2-amines (benzo[4,5]imidazo[1,2-*a*][1,3,5]triazines). For this purpose, 1-phenyl-3-aryl-1*H*-pyrazole-4-carbaldehydes **11a-c** were chosen as starting materials and were allowed to react with **3** in ethanol at reflux in the presence of a few drops of piperazine. Interestingly, benzo[4,5]imidazo[1,2-*a*][1,3,5]triazines **12a-c** which are linked to a pyrazole moiety at position-4 has been generated in good yields (Scheme 5).

The chemical composition of the products was verified by the different spectral tools. For example, the mass spectrum of **12b** (Ar = 4-MeC_6_H_4_) demonstrated a molecular ion peak at *m/z* 419 which fits with the proposed structure. The IR spectrum of compound **12** showed absorption bands at $\:\stackrel{-}{\nu\:}$ 3546 and 3325 cm^− 1^ for NH_2_ and NH stretching vibration. The ^1^H NMR indicated the presence of a signal at δ 2.24 ppm for methyl protons, a doublet at δ 6.57 ppm for *H*-4, a characteristic signal at δ 9.03 ppm for pyrazole-*H*5, and two singlet signals at δ 7.04 (2 H), 9.48 (1H) ppm for N*H*_2_ and N*H*, respectively. Signals for the aromatic protons appear at their appropriate position. The ^13^C NMR spectrum showed characteristic peaks at δ 20.9 and 59.9 ppm for methyl and C-4, respectively. Peaks for other carbons appear in their expected position.

**Scheme 6:**
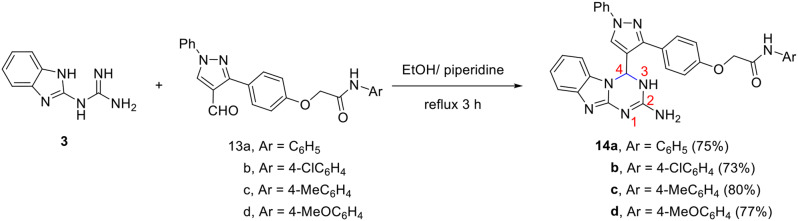
Synthesis of 1,3,4a,9-tetraza-4*H*-fluoren-2-amines **14a-d** that are linked to 2-(4-(1-phenyl-1*H*-pyrazol-3-yl)phenoxy)-*N*-arylacetamide moieties

Stimulated by the previously mentioned results, we broadened the scope of this reaction to include the synthesis of 1,3,4a,9-tetraza-4*H*-fluoren-2-amines **14a-d** that are linked to 2-(4-(1-phenyl-1*H*-pyrazol-3-yl)phenoxy)-*N*-arylacetamide moieties at position-4 in good yields by the direct reaction of the appropriate 2-(4-(4-formyl-1-phenyl-1*H*-pyrazol-3-yl)phenoxy)-*N*-arylacetamides **13a-d** with the corresponding mole equivalent of **3** (Scheme 6).

The chemical structure of the resulting compounds **14a-d** was confirmed based on spectral analyses. For instance, the mass spectrum of **14a** indicated a molecular-ion peak at *m/z* 554 which is relative to the proposed structure. The IR spectrum showed characteristic absorption bands at $\:\stackrel{-}{\nu\:}$ 3549, 3325, 1674 cm^− 1^ for NH_2_, NH, and amide C = O, respectively. The ^1^H NMR spectrum displayed two characteristic singlets at δ 4.79 and 9.08 ppm for OC*H*_2_ and pyrazole-*H*5, respectively. It showed further multiplets at δ 6.93–7.99 ppm integrated for 21 protons for the amino group, *H*-4, and aromatic protons, as well as two D_2_O-exchangeable singlets at δ 8.67 and 10.12 ppm for the two N*H* groups. The ^13^C NMR spectrum shows peaks at δ 67.1 and 166.5 ppm for O*C*H_2_ and amide *C* = O. Other peaks are present in close agreement with the proposed structure.

### Antibacterial activity

**Fig. 4:**
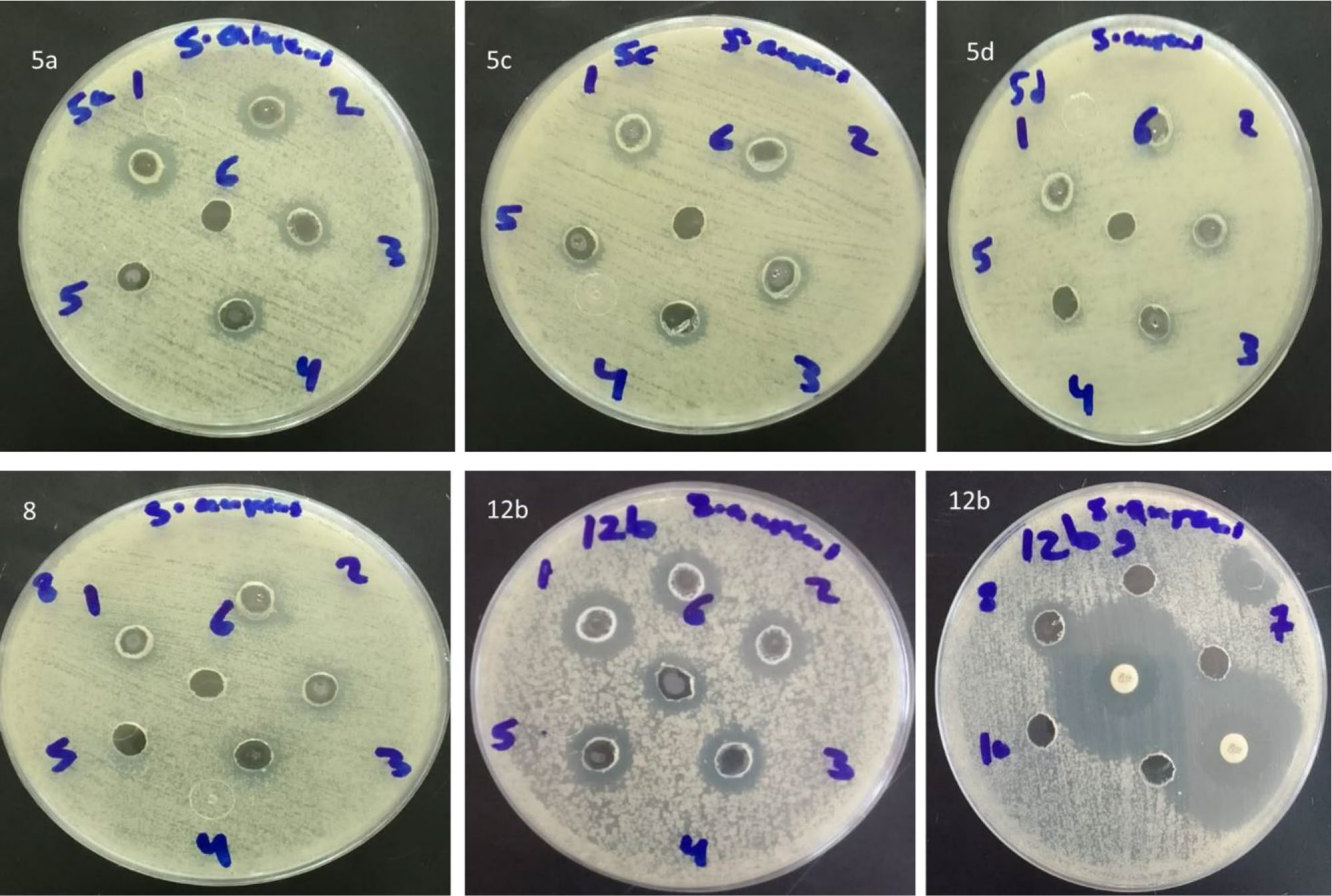
Agar well diffusion assay for estimating the MIC of compounds **5a, 5c, 5d, 8** and **12b** against *S. aureus*. The range of serial dilution concentrations was 1 = 10 mg/mL to 10 = 0.0195 mg/mL. CN10 means Gentamycin positive control

Agar well diffusion assay was used to assess the antibacterial activity of all the prepared compounds against *Staphylococcus aureus* (*S. aureus*) and *Bacillus subtilis* (*B. subtilis*) (gram-positive bacteria), *Escherichia coli* (*E. coli*) and *Pseudomonas aeruginosa* (*P. aeruginosa*) (gram-negative bacteria). The results showed that compounds **5a, 5d**, and **12b** were promising against *S. aureus* with the inhibition zones of 13 ± 1.4, 13 ± 2.1, and 16 ± 0.7 mm, respectively, and the most effective one was compound **12b** compared to Gentamycin (15 ± 0 mm) (Table 1). Concerning *B. subtilis*, compounds **5a, 5d, 12a**, and **12b** showed moderate activity with the inhibition zones of 14 ± 0, 16 ± 0.7, 14 ± 0, and 14 ± 1.4 mm, respectively, compared to Gentamycin (20 ± 1.4). Compound **5d** had the strongest action against *P. aeruginosa* (16 ± 0.7 mm), compared to Gentamycin (21 ± 0 mm). Compounds **5a, 5c**, and **12b** had moderate activity, whereas compound **14d** had the lowest activity (10 ± 0 mm) against *P. aeruginosa* compared to Gentamycin. All the synthesized compounds had no action against *E. coli*. So, the most promising results were shown against *S. aureus* compared to Gentamycin. Therefore, *S.aureus* strain was chosen for further MIC evaluation. Tables 2 and 3; Fig. 4 showed the results of MIC determination. It was found that compound **12b** had the lowest MIC value (78.1 µg/mL).

**Table 1:** Antibacterial activity of the synthesized compounds at a concentration of 10 mg/mL. The data was mean ± standard deviation (SD) of two separate experiments performed in triplicats

Inhibition zone diameter (mm) ± SD at 10 mg/mL				
Sample	S. aureus	B. subtilis	E. coli	*P*. aeruginosa
**5a**	13 ± 1.4	14 ± 0	NA	14 ± 0
**5b**	NA	NA	NA	NA
**5c**	12 ± 0	NA	NA	13 ± 1.4
**5d**	13 ± 2.1	16 ± 0.7	NA	16 ± 0.7
**8**	10 ± 0	NA	NA	NA
**12a**	NA	14 ± 0	NA	NA
**12b**	16 ± 0.7	14 ± 1.4	NA	14 ± 1.4
**12c**	NA	NA	NA	NA
14a	NA	NA	NA	NA
**14b**	NA	NA	NA	NA
**14c**	NA	NA	NA	NA
**14d**	NA	NA	NA	10 ± 0
Gentamycin	15 ± 0	20 ± 1.4	20 ± 2.1	21 ± 0
5% DMSO	0.0	0.0	0.0	0.0

(Gentamycin, 10 µg/disc) was positive control for gram-positive and gram-negative bacteria. *NA: No activity

**Table 2:** The effect of different concentrations (10-0.0195 mg/mL) of **5a, 5c, 5d, 8**, and **12b** on *S. Aureus*. The data was the mean of duplicate results ± standard deviation (SD)

Concentration (mg/mL)	S. aureus (Inhibition zone (mm) ± SD)				
	5a	5c	5d	8	12b
10	14 ± 1.4	12 ± 0	12.5 ± 2.1	10 ± 0	15.5 ± 0.7
5	13 ± 0.7	11 ± 0	10 ± 0.7	10 ± 0	14 ± 0
2.5	12 ± 0	11 ± 0	10 ± 0	9 ± 0.7	13 ± 1
1.25	11 ± 0	10 ± 0.7	9 ± 0	9 ± 0.7	13 ± 0
0.625	0	9 ± 0	0	0	12 ± 1
0.3125	0	0	0	0	12 ± 0.8
0.1563	0	0	0	0	11.5 ± 0
0.0781	0	0	0	0	11 ± 0
0.0391	0	0	0	0	0
0.0195	0	0	0	0	0

**Table 3:** Minimum inhibitory concentration (MIC) of compounds **5a, 5c, 5d, 8**, and **12b** against *S. Aureus*

Compound	S. aureus
	MIC (µg/mL)
**5a**	1250
**5c**	625
**5d**	1250
**8**	1250
**12b**	78.1

### Structure-activity relationship

**Fig. 5:**
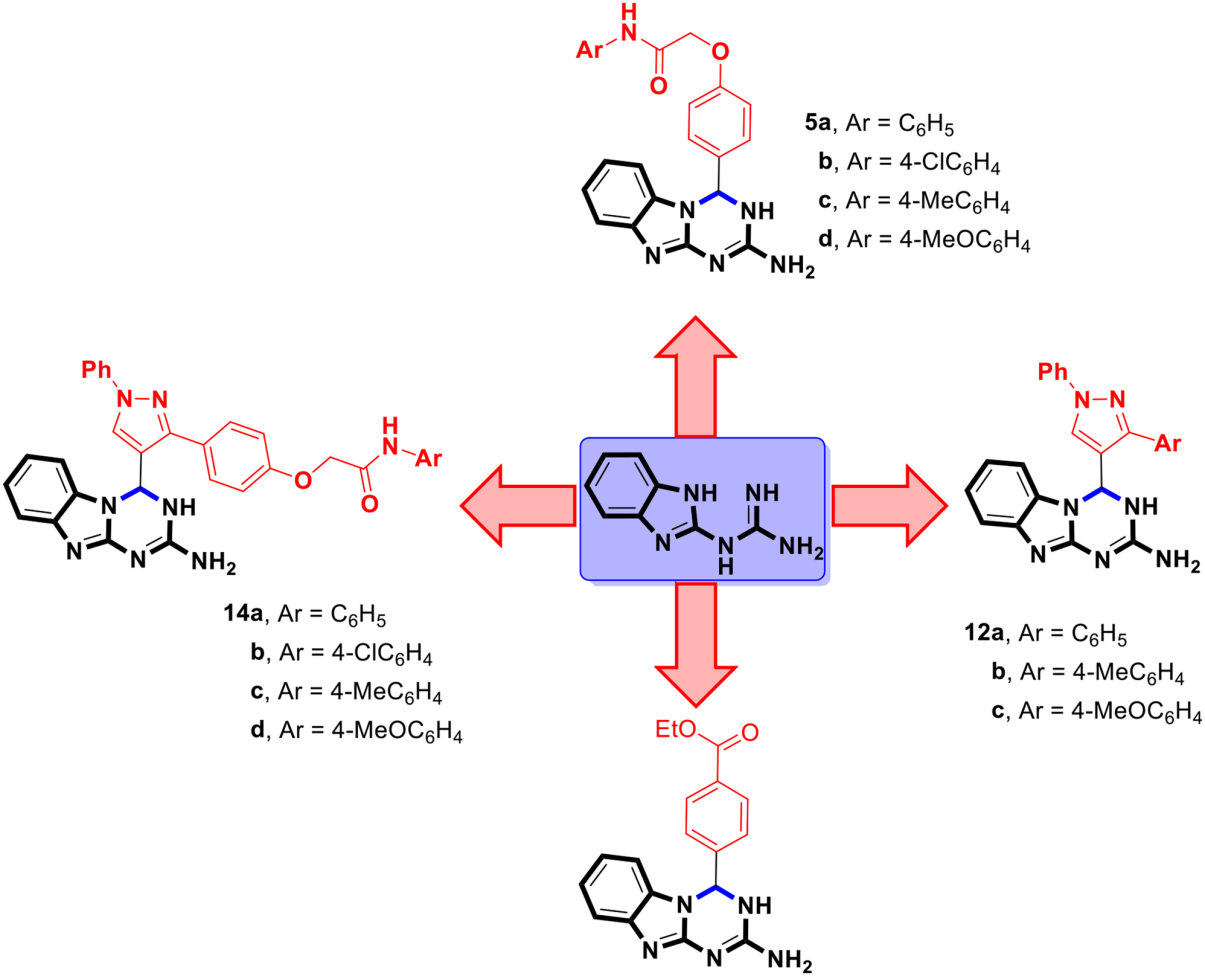
A general structure of the prepared benzo[4,5]imidazo[1,2-*a*][1,3,5]triazines **5,12**, and **14**

Figure 5 shows a design for the prepared series of benzo[4,5]imidazo[1,2-*a*][1,3,5]triazines **5,12**, and **14**.

The structure-activity relationship demonstrated that the derivatives **5a-d** and **12a-c** performed better than the derivatives **14a-d**. Compound **5d**, which included a 4-methoxyphenyl group (an electron-donating group), had the highest antibacterial activity against *S*. *aureus,P. aeuroginosa*, and *B. subtilis* among the **5a-d** derivatives. Compound **5a**, which included an unsubstituted phenyl group, had a moderate action against *S. aureus,P. aeruginosa* and *B. subtilis*. The *p*-tolyl group (electron-donating group) in derivative **5c** demonstrated modest antibacterial activity against *S. aureus* and *P. aeruginosa*. On the other hand, derivative **5b** with 4-chlorophenyl moiety (electron-withdrawing group) reduced antibacterial efficacy against the tested bacterial strains. The unsubstituted phenyl group in **12a** demonstrated moderate activity against *B. subtilis*, whereas derivative **12b** with *p*-tolyl group exhibited promising action against *S. aureus* and moderate activity against *B. subtilis* and *P. aeruginosa*, respectively. Compound **12c**, on the other hand, had decreased activity against all the strains tested. Among compounds **14a-d**, only **14d** containing a 4-methoxyphenyl group was effective. It showed limited efficacy against *P. aeruginosa*.

## Conclusion

We have described an effective method for creating novel annelated 1,3,4a,9-tetraza-4*H*-fluoren-2-amines ring systems incorporating phenoxy-*N*-arylacetamide, and pyrazole moieties. The reaction involves reacting one-mole equivalent of 1-(1*H*-benzo[*d*]imidazol-2-yl)guanidine with one-mole equivalent of the appropriate aldehydes. We concluded that compound **5d**’s promising action against *S. aureus* and *P. aeruginosa*, as well as its modest activity against *B. subtilis*, might be attributed to its inhibition of the MurG enzyme, as indicated by our molecular docking studies. Furthermore, the promising efficacy of compounds **5a** and **5c** against *S. aureus* might be attributed to their inhibitory action on bacterial tyrosyl tRNA synthetase and MurG.

### Supplementary file

Compounds **5a** is represented here as a representative example. Full experimental details of all compounds and spectral data are represented in the supplementary file.

### “Experimental

“Melting points were measured with using a Stuart melting point apparatus and were uncorrected. The IR spectra were recorded using Vector 22 FTIR-spectrophotometer (Brucker, Germany) as KBr pellets. The ^1^H and ^13^C NMR spectra were recorded in DMSO-*d*_*6*_ as a solvent with Mercury VXR-300 NMR spectrometer (Varian, USA) operating at 300 MHz and 75 MHz, using TMS as an internal standard. Chemical shifts were reported as *δ* values in ppm. Mass spectra were recorded with GCMS-QP-1000 EX mass spectrometer (Shimadzu, Japan) in EI (70 eV) model. The elemental analyses were performed using CHNS-932 Vario elemental analyzer (LECO, USA) at the Micro Analytical Centre, Cairo University”.

### General method for the synthesis of compounds 5a-d, 8, 12a-c and 14a-d

“A solution of 1-(1*H*-benzo[*d*]imidazol-2-yl)guanidine (**3**) (175 mg, 1 mmol) and the appropriate aldehyde (**4a-d**), (**6a-d), (11a-c), or (13a-d)** (1 mmol) in ethanol (10 mL) containing piperidine (2 drops) was heated at reflux for 3 h. The reaction mixture is allowed to cool to ambient temperature. A precipitate is formed which is subsequently filtered, washed with ethanol, and recrystallized from EtOH\dioxane (3:1, v/v) mixture to give the titled compound”.

### 2-(4-(2-Amino-3,4-dihydrobenzo[4,5]imidazo[1,2-*a*][1,3,5]triazin-4-yl)phenoxy)-*N*-phenylacetamide (5a)

Colorless powder (350 mg, 85%); Mp 235–237 ºC; IR (KBr): $\:\stackrel{-}{\nu\:}$ 3449 (N*H*_2_), 3325 (2N*H)*, 1674 (C = O), 1605 (C = N), 1520 (C = C) cm^− 1^; ^1^H NMR (300 MHz, DMSO-*d*_*6*_) δ 4.69 (s, 2 H, OC*H*_2_), 6.39 (s, 2 H, N*H*_2_, D_2_O exchangeable), 6.70 (d, *J* = 6.9 Hz, 2 H, Ar-*H*), 6.78 (t, *J* = 7.5 Hz, 1H, Ar-*H*), 6.93 (t, *J* = 7.5 Hz, 1H, Ar-*H*), 7.02 (d, *J* = 8.6 Hz, 2 H, Ar-*H*), 7.08 (d, *J* = 8.4 Hz, 1H, *H*4), 7.22 (d, *J* = 8.0 Hz, 1H, Ar-*H*), 7.26–7.36 (m, 4 H, Ar-*H*), 7.61 (d, *J* = 8.6 Hz, 2 H), 7.98 (br s, 1H, N*H*, D_2_O exchangeable), 10.06 (s, 1H, N*H*, D_2_O exchangeable) ppm; ^13^C NMR (75 MHz, DMSO-*d*_*6*_): δ 65.6, 67.1, 108.4, 115.0, 115.9, 119.0, 119.8, 120.9, 123.7, 127.8, 128.8, 131.2, 133.2, 138.4, 143.1, 153.5, 155.4, 158.4, 166.4 ppm; MS (EI, 70 eV): *m/z* (%) 412 ]M^+^[; Anal. Calcd for C_23_H_20_N_6_O_2_: C, 66.98; H, 4.89; N, 20.38. Found: C, 66.83; H, 4.70; N, 20.18%.

### 2-(4-(2-Amino-3,4-dihydrobenzo[4,5]imidazo[1,2-*a*][1,3,5]triazin-4-yl)phenoxy)-*N*-(4-chlorophenyl)acetamide (5b)

Pale yellow powder (370 mg, 83%); Mp 200–202 ºC; IR (KBr): $\:\stackrel{-}{\nu\:}$ 3448 (N*H*_2_), 3320 (2N*H)*, 1670 (C = O), 1600 (C = N), 1519 (C = C) cm^− 1^; ^1^H NMR (300 MHz, DMSO-*d*_*6*_) δ 4.69 (s, 2 H, OC*H*_2_), 6.34 (s, 2 H, N*H*_2_, D_2_O exchangeable), 6.69 (d, *J* = 7.2 Hz, 2 H, Ar-*H*), 6.76 (t, *J* = 7.2 Hz, 1H, Ar-*H*), 6.91 (t, *J* = 7.5 Hz, 1H, Ar-*H*), 7.01 (d, *J* = 8.7 Hz, 2 H, Ar-*H*), 7.22 (d, *J* = 7.7 Hz, 1H, *H*4), 7.32 (d, *J* = 8.8 Hz, 2 H, Ar-*H*), 7.36 (d, *J* = 8.9 Hz, 2 H, Ar-*H*), 7.65 (d, *J* = 8.9 Hz, 2 H, Ar-*H*), 7.92 (s, 1H, N*H*, D_2_O exchangeable), 10.20 (s, 1H, N*H*) ppm; ^13^C NMR (75 MHz, DMSO-*d*_6_) δ 65.9, 67.3, 108.3, 114.6, 116.0, 118.6, 120.1, 120.7, 121.1, 128.1, 129.7, 131.7, 132.6, 133.4, 135.2, 143.4, 154.9, 158.8, 166.6 ppm; MS (EI, 70 eV): *m/z* (%) 446 ]M^+^[ Anal. Calcd for C_23_H_19_ClN_6_O_2_: C, 61.82; H, 4.29; N, 18.81. Found: C, 61.66; H, 4.11; N, 18.64%.

### 2-(4-(2-Amino-3,4-dihydrobenzo[4,5]imidazo[1,2-*a*][1,3,5]triazin-4-yl)phenoxy)-*N*-(*p*-tolyl)acetamide (5c)

Colorless powder (354 mg, 83%); Mp 204–206 ºC; IR (KBr): $\:\stackrel{-}{\nu\:}$ 3448 (N*H*_2_), 3328 (2N*H)*, 1677 (C = O), 1609 (C = N), 1520 (C = C) cm^− 1^; ^1^H NMR (300 MHz, DMSO-*d*_*6*_) δ 2.25 (s, 3 H, C*H*_3_), 4.66 (s, 2 H, OC*H*_2_), 6.38 (s, 2 H, N*H*_2_, D_2_O exchangeable), 6.69 (d, *J* = 6.6 Hz, 2 H, Ar-*H*), 6.76 (t, *J* = 7.3 Hz, 1H, Ar-*H*), 6.92 (t, *J* = 7.1 Hz, 1H, Ar-*H*), 7.01 (d, *J* = 8.1 Hz, 2 H, Ar-*H*), 7.11 (d, *J* = 7.6 Hz, 2 H, Ar-*H*), 7.21 (d, *J* = 7.6 Hz, 1H, *H*4), 7.33 (d, *J* = 8.2 Hz, 2 H), 7.49 (d, *J* = 7.8 Hz, 2 H, Ar-*H*), 7.96 (br s, 1H, N*H*, D_2_O exchangeable), 9.96 (s, 1H, N*H*, D_2_O exchangeable) ppm; ^13^C NMR (75 MHz, DMSO-*d*_*6*_): δ 20.5, 65.5, 67.1, 108.3, 115.0, 115.9, 118.9, 119.9, 120.5, 120.8, 127.7, 129.1, 131.3, 132.7, 133.2, 135.9, 143.4, 155.4, 158.4, 166.1 ppm; MS (EI, 70 eV): *m/z* (%) 426 ]M^+^[ Anal. Calcd for C_24_H_22_N_6_O_2_: C, 67.59; H, 5.20; N, 19.71. Found: C, 67.39; H, 5.02; N, 19.53%.

### 2-(4-(2-Amino-3,4-dihydrobenzo[4,5]imidazo[1,2-*a*][1,3,5]triazin-4-yl)phenoxy)-*N*-(4-methoxyphenyl)acetamide (5d)

Grey powder (380 mg, 86%); Mp 240–242 ºC; IR (KBr): $\:\stackrel{-}{\nu\:}$ 3448 (N*H*_2_), 3330 (2N*H)*, 1678 (C = O), 1608 (C = N), 1522 (C = C) cm^− 1^; ^1^H NMR (300 MHz, DMSO-*d*_*6*_) δ 3.72 (s, 3 H, OC*H*_3_), 4.65 (s, 2 H, OC*H*_2_), 6.37 (s, 2 H, N*H*_2_, D_2_O exchangeable), 6.69 (d, *J* = 6.2 Hz, 2 H, Ar-*H*), 6.77 (t, *J* = 7.5 Hz, 1H, Ar-*H*), 6.88 (d, *J* = 8.2 Hz, 2 H, Ar-*H*), 6.93 (d, *J* = 7.5 Hz, 1H, Ar-*H*), 7.02 (d, *J* = 7.9 Hz, 2 H, Ar-*H*), 7.21 (d, *J* = 7.7 Hz, 1H, *H*4), 7.33 (d, *J* = 8.1 Hz, 2 H, Ar-*H*), 7.51 (d, *J* = 8.7 Hz, 2 H, Ar-*H*), 7.95 (s, 1H, N*H*, D_2_O exchangeable), 9.91 (s, 1H, N*H*, D_2_O exchangeable) ppm; ^13^C NMR (75 MHz, DMSO-*d*_6_) δ 58.1, 65.5, 69.1, 108.3, 115.0, 115.5, 116.0, 119.8, 120.1, 120.8, 129.1, 131.7, 132.3, 133.4, 135.9, 144.3, 154.5, 158.4, 160.3, 167.4 ppm; MS (EI, 70 eV): *m/z* (%) 442 ]M^+^[ Anal. Calcd for C_24_H_22_N_6_O_3_: C, 65.15; H, 5.01; N, 18.99. Found: C, 64.97; H, 4.87; N, 18.82%.

### Ethyl 4-(2-amino-3,4-dihydrobenzo[4,5]imidazo[1,2-*a*][1,3,5]triazin-4-yl)benzoate (8)

Wite powder (295 mg, 88%); Mp 208–210 ºC; IR (KBr): h$\:\stackrel{-}{\nu\:}$ 3418 (N*H*_2_), 3248 (N*H)*, 1713 (C = O), 1628 (C = N), 1528 (C = C) cm^− 1^; ^1^H NMR (300 MHz, DMSO-*d*_*6*_) δ 1.28 (t, *J* = 7.2 Hz, 3 H, COOCH_2_C*H*_3_), 4.29 (q, *J* = 7.2 Hz, 2 H, COOC*H*_2_CH_3_), 6.42 (s, 2 H, N*H*_2_, D_2_O exchangeable), 6.74 (d, *J* = 7.5 Hz, 1H, *H*4), 6.79 (t, *J* = 7.4 Hz, 1H, Ar-*H*), 6.84 (s, 1H, *H*4), 6.94 (t, *J* = 7.4 Hz, 1H, Ar-*H*), 7.24 (d, *J* = 7.8 Hz, 1H, Ar-*H*), 7.48 (d, *J* = 7.9 Hz, 2 H, Ar-*H*), 7.96 (d, *J* = 7.7 Hz, 2 H, Ar-*H*), 8.09 (s, 1H, N*H*, D_2_O exchangeable) ppm; ^13^C NMR (75 MHz, DMSO-*d*_*6*_): δ 14.2, 61.1, 65.4, 108.5, 116.1, 119.3, 121.4, 126.6, 128.7, 130.0, 130.7, 131.1, 143.2, 145.3, 155.2, 165.3 ppm; MS (EI, 70 eV): *m/z* (%) 335 ]M^+^[. Anal. Calcd for C_18_H_17_N_5_O_2_: C, 64.47; H, 5.11; N, 20.88. Found: C, 64.33; H, 4.95; N, 20.70%.

### 4-(1,3-Diphenyl-1*H*-pyrazol-4-yl)-3,4-dihydrobenzo[4,5]imidazo[1,2-*a*][1,3,5]triazin-2-amine (12a)

Colorless powder (352 mg, 87%); Mp 280–282 ºC; IR (KBr): $\:\stackrel{-}{\nu\:}$ 3540 (N*H*_2_), 3325 (N*H)*, 1604 (C = N), 1520 (C = C) cm^− 1^; ^1^H NMR (300 MHz, DMSO-*d*_*6*_) δ 6.40 (d, *J* = 7.9 Hz, 1H, *H*4), 6.51(s, 2 H, N*H*_2_, D_2_O exchangeable), 6.69 (t, *J* = 7.6 Hz, 1H, Ar-*H*), 6.88 (d, *J* = 7.2 Hz, 2 H, Ar-*H*), 7.15 (d, *J* = 7.8 Hz, 1H, Ar-*H*), 7.33 (t, *J* = 7.4 Hz, 1H, Ar-*H*), 7.41 (d, *J* = 6.6 Hz, 2 H, Ar-*H*), 7.50 (t, *J* = 7.7 Hz, 3 H, Ar-*H*), 7.65 (d, *J* = 7.6 Hz, 2 H, Ar-*H*), 7.90 (d, *J* = 8.0 Hz, 2 H, Ar-*H*), 8.25 (br s, 1H, N*H*, D_2_O exchangeable), 8.76 (s, 1H, pyrazole*-H*5) ppm; ^13^C NMR (75 MHz, DMSO-*d*_*6*_): δ 62.6, 111.2, 118.1, 118.5, 119.2, 122.9, 123.8, 127.0, 128.0, 128.2, 128.9, 129.2, 129.4, 129.6, 130.2, 137.6, 139.5, 150.5, 151.4, 157.7 ppm; MS (EI, 70 eV): *m/z* (%) 405 ]M^+^[ Anal. Calcd for C_24_H_19_N_7_: C, 71.09; H, 4.72; N, 24.18. Found: C, 70.96; H, 4.56; N, 24.04%.

### 4-(1-Phenyl-3-(*p*-tolyl)-1*H*-pyrazol-4-yl)-3,4-dihydrobenzo[4,5]imidazo[1,2-*a*][1,3,5]triazin-2-amine (12b)

Wite powder (369 mg, 88%); Mp 295–297 ºC; IR (KBr): h$\:\stackrel{-}{\nu\:}$ 3546 (N*H*_2_), 3325 (N*H)*, 1604 (C = N), 1525 (C = C) cm^− 1^; ^1^H NMR (300 MHz, DMSO-*d*_*6*_) δ 2.34 (s, 3 H, C*H*_3_), 6.58 (d, *J* = 7.9 Hz, 1H, *H*4), 7.04 (s, 2 H, N*H*_2_, D_2_O exchangeable), 7.17 (d, *J* = 7.8 Hz, 1H, Ar-*H*), 7.23 (d, *J* = 7.6 Hz, 2 H, Ar-*H*), 7.33 (d, *J* = 7.9 Hz, 3 H, Ar-*H*), 7.43 (d, *J* = 8.0 Hz, 2 H, Ar-*H*), 7.52 (t, *J* = 7.7 Hz, 3 H, Ar-*H*), 7.89 (d, *J* = 8.2 Hz, 2 H, Ar-*H*), 9.03 (s, 1H, pyrazole*-H*5), 9.48 (br s, 1H, N*H*, D_2_O exchangeable) ppm; ^13^C NMR (75 MHz, DMSO-*d*_*6*_): δ 20.9, 59.9, 110.2, 111.7, 118.5, 119.2, 122.9, 123.8, 127.0, 128.0, 128.2, 128.9, 129.2, 129.4, 129.7, 130.1, 138.2, 139.0, 150.8, 151.2, 156.7 ppm; MS (EI, 70 eV): *m/z* (%) 419 ]M^+^[ Anal. Calcd for C_25_H_21_N_7_: C, 71.58; H, 5.05; N, 23.37. Found: C, 71.43; H, 4.95; N, 23.18%.

### 4-(3-(4-Methoxyphenyl)-1-phenyl-1*H*-pyrazol-4-yl)-3,4-dihydrobenzo[4,5]imidazo[1,2-*a*][1,3,5]triazin-2-amine (12c)

Colorless powder (374 mg, 86%); Mp 287–289 ºC; IR (KBr): $\:\stackrel{-}{\nu\:}$ 3549 (N*H*_2_), 3325 (N*H)*, 1674 (C = O), 1605 (C = N), 1528 (C = C) cm^− 1^; ^1^H NMR (300 MHz, DMSO-*d*_*6*_) δ 3.78 (s, 3 H, OC*H*_3_), 6.50 (d, *J* = 7.6 Hz, 1H, *H*4), 6.92 (s, 2 H, N*H*_2_, D_2_O exchangeable), 6.97 (d, *J* = 8.3 Hz, 2 H, Ar-*H*), 7.07 (d, *J* = 7.5 Hz, 1H, Ar-*H*), 7.13 (s, 2 H, Ar-*H*), 7.27 (d, *J* = 7.8 Hz, 1H, Ar-*H*), 7.35 (d, *J* = 8.1 Hz, 1H, Ar-*H*), 7.50 (d, *J* = 6.1 Hz, 4 H, Ar-*H*), 7.88 (d, *J* = 8.1 Hz, 2 H, Ar-*H*), 8.37 (s, 1H, N*H*, D_2_O exchangeable), 8.87 (s, 1H, pyrazole-*H*5) ppm; ^13^C NMR (75 MHz, DMSO-*d*_*6*_): δ 55.4, 61.8, 110.5, 111.9, 118.4, 119.2, 122.9, 123.8, 127.2, 128.16, 128.18, 128.9, 129.3, 129.4, 129.7, 138.0, 138.6, 150.6, 151.2, 156.7, 159.4 ppm; MS (EI, 70 eV): *m/z* (%) 435 ]M^+^[ Anal. Calcd for C_25_H_21_N_7_O: C, 68.95; H, 4.86; N, 22.51. Found: C, 68.79; H, 4.69; N, 22.39%.

### 2-(4-(4-(2-Amino-3,4-dihydrobenzo[4,5]imidazo[1,2-*a*][1,3,5]triazin-4-yl)-1-phenyl-1*H*-pyrazol-3-yl)phenoxy)-*N*-phenylacetamide (14a)

White powder (415 mg, 75%); Mp 240–242 ºC; IR (KBr): $\:\stackrel{-}{\nu\:}$ 3549 (N*H*_2_), 3325 (N*H)*, 1674 (C = O), 1605 (C = N), 1528 (C = C) cm^− 1^; ^1^H NMR (300 MHz, DMSO-*d*_*6*_): δ 4.79 (s, 2 H, OC*H*_2_), 6.92 (s, 1H, *H*4), 7.09–7.18 (m, 3 H, N*H*_2_, Ar-*H*), 7.35 (s, 4 H, Ar-*H*), 7.54 (s, 4 H, Ar-*H*), 7.67 (d, *J* = 18.5 Hz, 5 H, Ar-*H*), 7.99 (s, 4 H, Ar-*H*), 8.67 (s, 1H, N*H*, D_2_O exchangeable), 9.08 (s, 1H, pyrazole*-H*5), 10.12 (s, 1H, N*H*, D_2_O exchangeable) ppm; ^13^C NMR (75 MHz, DMSO-*d*_*6*_): δ 67.2, 71.5, 110.5, 115.1, 115.7, 116.3, 119.0, 119.8, 120.6, 122.5, 123.9, 124.9, 127.3, 128.6, 128.9, 129.8, 130.0, 130.3, 138.4, 139.0, 142.8, 152.7, 153.6, 158.4, 160.2, 166.5.ppm; MS (EI, 70 eV): *m/z* (%) 554 ]M^+^[ Anal. Calcd for C_32_H_26_N_8_O_2_: C, 69.30; H, 4.73; N, 20.20. Found: C, 69.12; H, 4.57; N, 20.01%.

### 2-(4-(4-(2-Amino-3,4-dihydrobenzo[4,5]imidazo[1,2-*a*][1,3,5]triazin-4-yl)-1-phenyl-1*H*-pyrazol-3-yl)phenoxy)-*N*-(4-chlorophenyl)acetamide (14b)

Yellow powder (430 mg, 73%); Mp 180–182 ºC; IR (KBr): $\:\stackrel{-}{\nu\:}$ 3549 (N*H*_2_), 3325 (N*H)*, 1670 (C = O), 1604 (C = N), 1525 (C = C) cm^− 1^; ^1^H NMR (300 MHz, DMSO-*d*_*6*_): δ 4.79 (s, 2 H, OC*H*_2_), 6.59 (d, *J* = 8.8 Hz, 1H, *H*4), 7.12 (t, *J* = 8.9 Hz, 3 H, N*H*_2_, Ar-*H*), 7.39 (td, *J* = 12.3, 8.3 Hz, 5 H, Ar-*H*), 7.55 (d, *J* = 7.9 Hz, 2 H, Ar-*H*), 7.58–7.66 (m, 2 H, Ar-*H*), 7.69 (d, *J* = 8.8 Hz, 2 H, Ar-*H*), 7.95 (dd, *J* = 12.7, 8.6 Hz, 5 H, Ar-*H*), 9.27 (s, 1H, pyrazole*-H*5), 9.97 (s, 1H, N*H*, D_2_O exchangeable), 10.28 (s, 1H, N*H*, D_2_O exchangeable) ppm; ^13^C NMR (75 MHz, DMSO-*d*_6_): δ 66.9, 70.7, 108.9, 114.86, 114.93, 115.4, 115.9, 118.3, 118.9, 119.2, 119.9, 121.1, 125.0, 126.6, 129.1, 129.4, 129.5, 130.3, 131.1, 133.5, 135.8, 138.6, 143.3, 150.3, 158.1, 166.3 ppm;.MS (EI, 70 eV): *m/z* (%) 589 ]M^+^[ Anal. Calcd for C_32_H_25_ClN_8_O_2_: C, 65.25; H, 4.28; N, 19.02. Found: C, 65.05; H, 4.15; N, 18.86%.

### 2-(4-(4-(2-Amino-3,4-dihydrobenzo[4,5]imidazo[1,2-*a*][1,3,5]triazin-4-yl)-1-phenyl-1*H*-pyrazol-3-yl)phenoxy)-*N*-(*p*-tolyl)acetamide (14c)

Colorless powder (454 mg, 80%); Mp 193–195 ºC; IR (KBr): $\:\stackrel{-}{\nu\:}$ 3549 (N*H*_2_), 3325 (N*H)*, 1672 (C = O), 1604 (C = N), 1527 (C = C) cm^− 1^; ^1^H NMR (300 MHz, DMSO-*d*_*6*_) δ 2.26 (s, 3 H, C*H*_3_), 4.72 (s, 2 H, OC*H*_2_), 6.29 (s, 2 H, N*H*_2_, D_2_O exchangeable), 6.41 (d, *J* = 7.8 Hz, 1H, *H*4), 6.79–6.92 (m, 3 H, Ar-*H*), 7.06–7.20 (m, 5 H, Ar-*H*), 7.30–7.35 (m, 1H, Ar-*H*), 7.46–7.55 (m, 5 H, Ar-*H*), 7.64 (d, *J* = 7.2 Hz, 1H, Ar-*H*), 7.90 (d, *J* = 9.1 Hz, 2 H, Ar-*H*), 8.70 (s, 1H, N*H*, D_2_O exchangeable), 9.15 (s, 1H, pyrazole*-H*5), 10.02 (s, 1H, N*H*, D_2_O exchangeable) ppm; ^13^C NMR (75 MHz, DMSO-*d*_*6*_): δ 20.5, 67.2, 70.7, 108.4, 114.8, 114.9, 115.9, 118.3, 119.0, 119.2, 119.8, 120.8, 125.2, 126.6, 129.1, 129.5, 129.6, 129.7, 130.1, 131.5, 132.7, 135.8, 139.1, 143.3, 150.4, 158.0, 166.1 ppm; MS (EI, 70 eV): *m/z* (%) 568 ]M^+^[ Anal. Calcd for C_33_H_28_N_8_O_2_: C, 69.70; H, 4.96; N, 19.71. Found: C, 69.59; H, 4.81; N, 19.58%.

### 2-(4-(4-(2-Amino-3,4-dihydrobenzo[4,5]imidazo[1,2-*a*][1,3,5]triazin-4-yl)-1-phenyl-1*H*-pyrazol-3-yl)phenoxy)-*N*-(4-methoxyphenyl)acetamide (14d)

Grey powder (450 mg, 77%); Mp 182–184 ºC; IR (KBr): $\:\stackrel{-}{\nu\:}$ 3549 (N*H*_2_), 3325 (N*H)*, 1674 (C = O), 1605 (C = N), 1528 (C = C) cm^− 1^; ^1^H NMR (300 MHz, DMSO-*d*_*6*_) δ 3.72 (s, 3 H, OC*H*_3_), 4.70 (s, 2 H, OC*H*_2_), 6.30 (s, 2 H, N*H*_2_, D_2_O exchangeable), 6.41 (d, *J* = 7.3 Hz, 1H, *H*4), 6.70 (d, *J* = 7.6 Hz, 1H, Ar-*H*), 6.88 (t, *J* = 4.5 Hz, 4 H, Ar-*H*), 7.04–7.11 (m, 2 H, Ar-*H*), 7.44–7.68 (m, 8 H, Ar-*H*), 7.89 (d, *J* = 8.2 Hz, 2 H, Ar-*H*), 8.70 (s, 1H, N*H*, D_2_O exchangeable), 9.28 (s, 1H, pyrazole*-H*5), 9.95 (s, 1H, N*H*, D_2_O exchangeable) ppm; MS (EI, 70 eV): *m/z* (%) 584 ]M^+^[ Anal. Calcd for C_33_H_28_N_8_O_3_: C, 67.80; H, 4.83; N, 19.17. Found: C, 67.61; H, 4.67; N, 18.99%.

### Antibacterial assay

The antibacterial activity of the prepared compounds was assessed by using the agar well diffusion assay. The antibacterial activity was screened against two gram-positive bacteria *Staphylococcus aureus* (ATCC 6538) and *Bacillus subtilis* (DSM 1088) as well as two gram-negative bacteria *Pseudomonas aeruginosa* (ATCC 10145) and *Escherichia coli* (ATCC 8739). Dimethyl sulfoxide (DMSO) was used to make a solution of 10 mg/mL of each synthesized compound. The nutrient agar medium was poured on the plates and let to be cooled to 45 °C. 10^5^-10^6^ colony forming unit (CFU) per mL from an overnight bacterial culture was cultured on nutrient agar plates. Then, 6 mm wells were created in the nutritional medium using sterile metallic bores. After that, 20 µL of each tested compound (10 mg/mL) was added to the prepared well. Herein, the negative control was 5% DMSO and the results were compared to standard Gentamycin (10 µg/disc) and Clindamycin (2 µg/disc) (positive controls). The inhibition zone diameter in (mm) was measured using a calliper after the incubation of the plates at 37 °C for (18–24) hours. The experiment was repeated twice and in each time was done in triplicates. The minimum inhibition concentration (MIC) of compounds **5a, 5c, 5d, 8** and **12b** was determined against *S. aureus*. A serial dilution method with concentrations ranging from 10 to 0.0195 mg/mL was utilized. The MIC is the lowest concentration of the target drug that can inhibit the growth of the studied bacteria.

## Electronic supplementary material

Below is the link to the electronic supplementary material.

Supplementary Material 1

## Data Availability

All data generated or analyzed during this study are included in this published article and its supplementary information file. Data available at request (Ismail A. Abdelhamid, ismail_shafy@yahoo.com, ismail_shafy@cu.edu.eg).

## References

[1] Shrivastava N, Naim MJ, Alam MJ, Nawaz F, Ahmed S, Alam O. Benzimidazole Scaffold as Anticancer Agent: synthetic approaches and structure–activity relationship. Arch Pharm (Weinheim). 2017;350:e201700040. 10.1002/ARDP.201700040. 10.1002/ardp.201700040 28544162

[2] Akhtar MJ, Yar MS, Sharma VK, Khan AA, Ali Z, Haider MR, et al. Recent progress of Benzimidazole hybrids for Anticancer potential. Curr Med Chem. 2019;27:5970–6014. 10.2174/0929867326666190808122929 31393240

[3] Feng LS, Su WQ, Cheng JB, Xiao T, Li HZ, Chen DA, et al. Benzimidazole hybrids as anticancer drugs: an updated review on anticancer properties, structure–activity relationship, and mechanisms of action (2019–2021). Arch Pharm (Weinheim). 2022;355:2200051. 10.1002/ARDP.202200051. 10.1002/ardp.202200051 35385159

[4] Satija G, Sharma B, Madan A, Iqubal A, Shaquiquzzaman M, Akhter M, et al. Benzimidazole based derivatives as anticancer agents: structure activity relationship analysis for various targets. J Heterocycl Chem. 2022;59:22–66. 10.1002/JHET.4355.

[5] Zhou W, Zhang W, Peng Y, Jiang Z-H, Zhang L, Du Z. Design, synthesis and Anti-tumor Activity of Novel Benzimidazole-Chalcone hybrids as non-intercalative topoisomerase II catalytic inhibitors. Molecules. 2020;25. 10.3390/molecules25143180 PMC7397320 32664629

[6] Wu L, Yang Y, Wang Z, Wu X, Su F, Li M, et al. Design, synthesis, and biological evaluation of aromatic amide-substituted benzimidazole-derived chalcones. The effect of upregulating TP53 protein expression. Molecules. 2020;25:1162. 32150865 10.3390/molecules25051162 PMC7179225

[7] Çevik UA, Celik I, Işık A, Pillai RR, Tallei TE, Yadav R, et al. Synthesis, molecular modeling, quantum mechanical calculations and ADME estimation studies of benzimidazole-oxadiazole derivatives as potent antifungal agents. J Mol Struct. 2022;1252:132095.

[8] Güzel E, Acar Çevik U, Evren AE, Bostancı HE, Gül ÜD, Kayış U, et al. Synthesis of Benzimidazole-1,2,4-triazole derivatives as potential antifungal agents targeting 14α-Demethylase. ACS Omega. 2022;8:4369–84. 10.1021/ACSOMEGA.2C07755/SUPPL_FILE/AO2C07755_SI_001.PDF. 10.1021/acsomega.2c07755 PMC9893751 36743066

[9] Kanwal A, Ahmad M, Aslam S, Naqvi SAR, Saif MJ. Recent advances in antiviral benzimidazole derivatives: a Mini Review. Pharm Chem J. 2019;53:179–87. 10.1007/S11094-019-01976-3/FIGURES/11.

[10] Chen M, Su S, Zhou Q, Tang X, Liu T, Peng F, et al. Antibacterial and antiviral activities and action mechanism of flavonoid derivatives with a benzimidazole moiety. J Saudi Chem Soc. 2021;25:101194.

[11] Marinescu M. Benzimidazole-Triazole hybrids as Antimicrobial and Antiviral agents: a systematic review. Antibiot 2023. Page 1220. 2023;12:12:1220. 10.3390/ANTIBIOTICS12071220. 10.3390/antibiotics12071220 PMC10376251 37508316

[12] Wang XJ, Xi MY, Fu JH, Zhang FR, Cheng GF, Yin DL, et al. Synthesis, biological evaluation and SAR studies of benzimidazole derivatives as H1-antihistamine agents. Chin Chem Lett. 2012;23:707–10.

[13] Veerasamy R, Roy A, Karunakaran R, Rajak H. Structure–Activity Relationship Analysis of Benzimidazoles as Emerging Anti-Inflammatory Agents: An Overview. Pharm 2021, Vol 14, Page 663. 2021;14:663. 10.3390/PH14070663 10.3390/ph14070663 PMC8308831 34358089

[14] Vasava MS, Bhoi MN, Rathwa SK, Jethava DJ, Acharya PT, Patel DB, et al. Benzimidazole: a milestone in the field of Medicinal Chemistry. Mini Rev Med Chem. 2019;20:532–65. 10.2174/1389557519666191122125453 31755386

[15] Yang X, Syed R, Fang B, Zhou CH. A New Discovery towards Novel Skeleton of Benzimidazole-Conjugated pyrimidinones as Unique Effective Antibacterial agents. Chin J Chem. 2022;40:2642–54. 10.1002/CJOC.202200326.

[16] Ahmed Saleh Alzahrani S, Nazreen S, Elhenawy AA, Neamatallah T, Mahboob M, Synthesis. Biological evaluation, and Molecular Docking of New Benzimidazole-1,2,3-Triazole hybrids as Antibacterial and Antitumor agents. Polycycl Aromat Compd. 2023;43:3380–91. 10.1080/10406638.2022.2069133.

[17] Tiglani D, Salahuddin, Mazumder A, Kumar R, Yar MS, Ahsan MJ, et al. Synthesis anticonvulsant and cytotoxic evaluation of Benzimidazole-Quinoline hybrids Schiff Base Analogs. Polycycl Aromat Compd. 2023. 10.1080/10406638.2023.2183969.

[18] Shabana K, Salahuddin, Mazumder A, Singh H, Kumar R, Tyagi S, et al. Synthesis, characterization, in Silico and in vivo evaluation of Benzimidazole-Bearing Quinoline Schiff Bases as New Anticonvulsant agents. ChemistrySelect. 2023;8:e202300209. 10.1002/SLCT.202300209.

[19] Siwach A, Verma PK. Synthesis and therapeutic potential of imidazole containing compounds. BMC Chem. 2021;15:12. 33602331 10.1186/s13065-020-00730-1 PMC7893931

[20] Garcia-Quiroz J, Camacho J. Astemizole: an old anti-histamine as a New Promising Anti-cancer Drug. Anticancer Agents Med Chem. 2012;11:307–14. 10.2174/187152011795347513 21443504

[21] Chong CR, Chen X, Shi L, Liu JO, Sullivan DJ. A clinical drug library screen identifies astemizole as an antimalarial agent. Nat Chem Biol 2006 28. 2006;2:415–6. 10.1038/nchembio806. 10.1038/nchembio806 16816845

[22] Church MK, Labeaga L. Bilastine: a new H1-antihistamine with an optimal profile for updosing in urticaria. J Eur Acad Dermatology Venereol. 2017;31:1447–52. 10.1111/JDV.14305. 10.1111/jdv.14305 28467671

[23] Church MK. Safety and efficacy of bilastine: a new H1-antihistamine for the treatment of allergic rhinoconjunctivitis and urticaria. Expert Opin Drug Saf. 2011;10:779–93. 10.1517/14740338.2011.604029. 21831011 10.1517/14740338.2011.604029

[24] Reddy TP, Dussa N, Mamidi S, Panasa M, Chavakula R, Padma M. Identification and synthesis of potential impurities of bilastine drug substance. Chem Pap. 2022;76:4137–45. 10.1007/S11696-022-02157-5/SCHEMES/5.

[25] Iwamoto K, Uehara Y, Inoue Y, Taguchi K, Muraoka D, Ogo N, et al. Inhibition of STAT3 by Anticancer Drug Bendamustine. PLoS ONE. 2017;12:e0170709. 10.1371/JOURNAL.PONE.0170709. 28125678 10.1371/journal.pone.0170709 PMC5268383

[26] Lalic H, Aurer I, Batinic D, Visnjic D, Smoljo T, Babic A, Bendamustine. A review of pharmacology, clinical use and immunological effects (review). Oncol Rep. 2022;47:1–16. 10.3892/OR.2022.8325/HTML. 35506458 10.3892/or.2022.8325 PMC9100486

[27] Barman Balfour JA, Goa KL, Bendamustine. Drugs. 2001;61:631–8. 10.2165/00003495-200161050-00009/FIGURES/2. 11368287 10.2165/00003495-200161050-00009

[28] Singla P, Luxami V, Paul K. Triazine as a promising scaffold for its versatile biological behavior. Eur J Med Chem. 2015;102:39–57. 26241876 10.1016/j.ejmech.2015.07.037

[29] Ahmadi F, Mirzaei P, Bazgir A. Cobalt-catalyzed isocyanide insertion cyclization to dihydrobenzoimidazotriazins. Tetrahedron Lett. 2017;58:4281–4.

[30] Srivastava JK, Dubey P, Singh S, Bhat HR, Kumawat MK, Singh UP. Discovery of novel 1,3,5-triazine-thiazolidine-2,4-diones as dipeptidyl peptidase-4 inhibitors with antibacterial activity targeting the S1 pocket for the treatment of type 2 diabetes. RSC Adv. 2015;5:14095–102. 10.1039/C4RA16903D.

[31] Chauhan K, Sharma M, Shivahare R, Debnath U, Gupta S, Prabhakar YS, et al. Discovery of triazine mimetics as potent antileishmanial agents. ACS Med Chem Lett. 2013;4:1108–13. 10.1021/ML400317E/SUPPL_FILE/ML400317E_SI_001.PDF. 24900613 10.1021/ml400317e PMC4027224

[32] Khazir J, Mir BA, Chashoo G, Maqbool T, Riley D, Pilcher L. Design, synthesis, and anticancer evaluation of acetamide and hydrazine analogues of pyrimidine. J Heterocycl Chem. 2020;57:1306–18.

[33] Bhavsar D, Trivedi J, Parekh S, Savant M, Thakrar S, Bavishi A, et al. Synthesis and in vitro anti-HIV activity of N-1,3-benzo[d]thiazol-2-yl-2- (2-oxo-2H-chromen-4-yl)acetamide derivatives using MTT method. Bioorg Med Chem Lett. 2011;21:3443–6. 10.1016/j.bmcl.2011.03.105. 21515046 10.1016/j.bmcl.2011.03.105

[34] Gull Y, Rasool N, Noreen M, Altaf AA, Musharraf SG, Zubair M, et al. Synthesis of N-(6-arylbenzo[d]thiazole-2-acetamide derivatives and their biological activities: an experimental and computational approach. Molecules. 2016;21:1–17. 10.3390/molecules21030266 PMC6273329 26927044

[35] Ölgen S, Bakar F, Aydin S, Nebioǧlu D, Nebioǧlu S. Synthesis of new indole-2-carboxamide and 3-acetamide derivatives and evaluation their antioxidant properties. J Enzyme Inhib Med Chem. 2013;28:58–64. 22145595 10.3109/14756366.2011.631183

[36] Ang W, Lin YN, Yang T, Yang JZ, Pi WY, Yang YH, et al. Synthesis and biological evaluation of 2-(3-fluoro-4-nitro phenoxy)-N-phenylacetamide derivatives as novel potential affordable antitubercular agents. Molecules. 2012;17:2248–58. 10.3390/molecules17022248. 22357321 10.3390/molecules17022248 PMC6268079

[37] Yele V, Azam MA, Wadhwani AD, Synthesis. Molecular Docking and Biological evaluation of 2-Aryloxy-N-Phenylacetamide and N′-(2-Aryloxyoxyacetyl) Benzohydrazide Derivatives as potential Antibacterial agents. Chem Biodivers. 2021;18:e2000907. 33576162 10.1002/cbdv.202000907

[38] Mikhailovskii AG, Pogorelova ES, Pershina NN, Makhmudov RR, Novikova VV. Synthesis and analgesic, Antihypoxic, and antimicrobial activity of (Z)-2-(2-Arylhydrazono)-2-(3,3-Dimethyl-3,4-Dihydroisoquinolin-1-Yl)Acetamides. Pharm Chem J. 2020;53:1013–7.

[39] Yusov AS, Chashchina SV, Mikhailovskii AG, Rudakova IP. Synthesis and analgesic and anti-inflammatory activities of (3,3-Dipropyl-6,7-Dimethoxy-3,4-Dihydroisoquinolin-1(2H)-Ylidene)-Acetamide Hydrochlorides. Pharm Chem J. 2019;53:35–9.

[40] Severina HI, Skupa OO, Voloshchuk NI, Georgiyants VA. Synthesis, docking study, and pharmacological evaluation of S-acetamide derivatives of 4,6-dimethyl-2-thiopyrimidine as anticonvulsant agents. J Appl Pharm Sci. 2020;10:1–8.

[41] Mary SJJ, Siddique MUM, Pradhan S, Jayaprakash V, James C. Quantum chemical insight into molecular structure, NBO analysis of the hydrogen-bonded interactions, spectroscopic (FT–IR, FT–Raman), drug likeness and molecular docking of the novel anti COVID-19 molecule 2-[(4,6-diaminopyrimidin-2-yl)sulfanyl]-N-(4-fluo. Spectrochim Acta - Part Mol Biomol Spectrosc. 2021;244:118825. 10.1016/j.saa.2020.118825. 10.1016/j.saa.2020.118825 PMC7419267 32866803

[42] Borsoi AF, Paz JD, Abbadi BL, Macchi FS, Sperotto N, Pissinate K, et al. Design, synthesis, and evaluation of new 2-(quinoline-4-yloxy)acetamide-based antituberculosis agents. Eur J Med Chem. 2020;192:112179. 32113048 10.1016/j.ejmech.2020.112179

[43] Metwally NH, Badawy MA, Okpy DS. Synthesis and anticancer activity of some new thiopyrano[2,3-d]thiazoles incorporating pyrazole moiety. Chem Pharm Bull. 2015;63:495–503. 10.1248/cpb.c14-00885 26133066

[44] Alam R, Wahi D, Singh R, Sinha D, Tandon V, Grover A, et al. Design, synthesis, cytotoxicity, HuTopoIIα inhibitory activity and molecular docking studies of pyrazole derivatives as potential anticancer agents. Bioorg Chem. 2016;69:77–90. 27744115 10.1016/j.bioorg.2016.10.001

[45] Farghaly AA, Bekhit AA, Park JY. Design and synthesis of some oxadiazolyl, thiadiazolyl, thiazolidinyl, and thiazolyl derivatives of 1H-pyrazole as anti-inflammatory antimicrobial agents. Arch Pharm (Weinheim). 2000;333:53–7. 10.1002/(sici)1521-4184(200002)333:2/3<53::aid-ardp53>3.0.co;2-e 10783518

[46] Kendre BV, Landge MG, Bhusare SR. Synthesis and biological evaluation of some novel pyrazole, isoxazole, benzoxazepine, benzothiazepine and benzodiazepine derivatives bearing an aryl sulfonate moiety as antimicrobial and anti-inflammatory agents. Arab J Chem. 2015;12:2091–7.

[47] Viveka S, Dinesha, Madhu LN, Nagaraja GK. Synthesis of new pyrazole derivatives via multicomponent reaction and evaluation of their antimicrobial and antioxidant activities. Monatsh Chem. 2015;146:1547–55.

[48] Sallam HA, Elgubbi AS, El-Helw EAE. Synthesis and antioxidant screening of new 2-cyano-3-(1,3-diphenyl-1H-pyrazol-4-yl)acryloyl amide derivatives and some pyrazole-based heterocycles. Synth Commun. 2020;50:2066–77.

[49] Bellam M, Gundluru M, Sarva S, Chadive S, Netala VR, Tartte V, et al. Synthesis and antioxidant activity of some new N-alkylated pyrazole-containing benzimidazoles. Chem Heterocycl Compd. 2017;53:173–8.

[50] Kaushik D, Khan SA, Chawla G, Kumar S. N’-[(5-chloro-3-methyl-1-phenyl-1H-pyrazol-4-yl)methylene] 2/4-substituted hydrazides: synthesis and anticonvulsant activity. Eur J Med Chem. 2010;45:3943–9. 10.1016/J.EJMECH.2010.05.049. 20573423 10.1016/j.ejmech.2010.05.049

[51] Jain M, Sakhuja R, Khanna P, Bhagat S, Jain S. A facile synthesis of novel unsymmetrical bis-spiro– 2, 4’-diones. Arkivoc. 2008;xv:54–64. http://www.arkat-usa.org/get-file/25953/. Accessed 10 Aug 2015.

[52] Yang GY, Oh K-A, Park N-J, Jung Y-S. New oxime reactivators connected with CH2O(CH2)nOCH2 linker and their reactivation potency for organophosphorus agents-inhibited acetylcholinesterase. Bioorg Med Chem. 2007;15:7704–10. 10.1016/j.bmc.2007.08.056. 17869525 10.1016/j.bmc.2007.08.056

[53] Di Giacomo B, Bedini A, Spadoni G, Tarzia G, Fraschini F, Pannacci M, et al. Synthesis and biological activity of new melatonin dimeric derivatives. Bioorg Med Chem. 2007;15:4643–50. 10.1016/j.bmc.2007.03.080. 17481904 10.1016/j.bmc.2007.03.080

[54] Antonini I, Polucci P, Magnano A, Gatto B, Palumbo M, Menta E, et al. Design, synthesis, and biological properties of new bis(acridine-4-carboxamides) as anticancer agents. J Med Chem. 2003;46:3109–15. 10.1021/jm030820x. 12825949 10.1021/jm030820x

[55] Antonini I, Polucci P, Magnano A, Sparapani S, Martelli S. Rational design, synthesis, and biological evaluation of bis(pyrimido[5,6,1-de]acridines) and bis(pyrazolo[3,4,5-kl]acridine-5-carboxamides) as new anticancer agents. J Med Chem. 2004;47:5244–50. 10.1021/jm049706k. 15456268 10.1021/jm049706k

[56] Wang C, Jung G-Y, Hua Y, Pearson C, Bryce MR, Petty MC, et al. An efficient pyridine- and oxadiazole-containing hole-blocking material for Organic Light-Emitting diodes: synthesis, Crystal structure, and device performance. Chem Mater. 2001;13:1167–73. 10.1021/cm0010250.

[57] Wang C, Jung G-Y, Batsanov AS, Bryce MR, Petty MC. New electron-transporting materials for light emitting diodes: 1,3,4-oxadiazole–pyridine and 1,3,4-oxadiazole–pyrimidine hybrids. J Mater Chem. 2002;12:173–80. 10.1039/b106907c.

[58] He F, Li X, Huo C, Chu S, Cui Z, Li Y, et al. Evaluation of fluorene-caused ecotoxicological responses and the mechanism underlying its toxicity in Eisenia fetida: multi-level analysis of biological organization. J Hazard Mater. 2022;437:129342. 35716570 10.1016/j.jhazmat.2022.129342

[59] Lu H, Deng C, Yu Z, Zhang D, Li W, Huang J, et al. Synergistic degradation of fluorene in soil by dielectric barrier discharge plasma combined with P25/NH2-MIL-125(Ti). Chemosphere. 2022;296:133950. 35176305 10.1016/j.chemosphere.2022.133950

[60] Peiffer J, Grova N, Hidalgo S, Salquèbre G, Rychen G, Bisson JF, et al. Behavioral toxicity and physiological changes from repeated exposure to fluorene administered orally or intraperitoneally to adult male Wistar rats: a dose–response study. Neurotoxicology. 2016;53:321–33. 26616911 10.1016/j.neuro.2015.11.006

[61] Huisman HO. Approaches to total synthesis of Heterocyclic Steroidal systems. Angew Chem Int Ed. 1971;10:450–9. 10.1002/ANIE.197104501.

[62] Engel CR, Roy Chowdhury MN. Steroids and related products. XXVII. The synthesis of 11-oxa steroids. I. 11-oxaprogesterone. Tetrahedron Lett. 1968;9:2107–11. 10.1016/s0040-4039(00)89754-3 5646944

[63] Helmy MT, Sroor FM, Mahrous KF, Mahmoud K, Hassaneen HM, Saleh FM, et al. Anticancer activity of novel 3-(furan-2-yl)pyrazolyl and 3-(thiophen-2-yl)pyrazolyl hybrid chalcones: synthesis and in vitro studies. Arch Pharm (Weinheim). 2022;355:e2100381. 10.1002/ardp.202100381. 34939695 10.1002/ardp.202100381

[64] Kamel MG, Sroor FM, Othman AM, Mahrous KF, Saleh FM, Hassaneen HM, et al. Structure-based design of novel pyrazolyl–chalcones as anti-cancer and antimicrobial agents: synthesis and in vitro studies. Monatsh Chem. 2022;153:211–21. 10.1007/s00706-021-02886-5.

[65] Ibrahim YA, Abbas AA, Elwahy AHM. Selective synthesis and structure of 2-N-and 3-S-glucosyl-1, 2, 4-triazoles of potential biological interest. Carbohydr Lett. 1999;3:331–8.

[66] Mekky AEM, Elwahy AHM. Synthesis of Novel Benzo-substituted macrocyclic ligands containing thienothiophene subunits. J Heterocycl Chem. 2014;51:E34–41.

[67] Diab HM, Abdelhamid IA, Elwahy AHM. ZnO-Nanoparticles-Catalyzed synthesis of poly(tetrahydrobenzimidazo[2,1- b ]quinazolin-1(2 H)-ones) as novel multi-armed molecules. Synlett. 2018;29:1627–33.

[68] Abdella AM, Mohamed MF, Mohamed AF, Elwahy AHM, Abdelhamid IA. Novel bis(dihydropyrano[3,2-c]chromenes): synthesis, Antiproliferative Effect and Molecular Docking Simulation. J Heterocycl Chem. 2018;55:498–507.

[69] Abdelhamid IA. Synthesis of novel spiro cyclic 2-oxindole derivatives of 6-amino-4H-pyridazine via [3 + 3] atom combination utilizing chitosan as a catalyst. Synlett. 2009;:625–7.

[70] Hamed AA, Ali EA, Abdelhamid IA, Saad GR, Elsabee MZ. Synthesis of novel chitosan-Schiff bases nanoparticles for high efficiency Helicobacter pylori inhibition. Int J Biol Macromol. 2024;274:133499. 10.1016/J.IJBIOMAC.2024.133499. 38944085 10.1016/j.ijbiomac.2024.133499

[71] Abdelwahab RE, Ragheb MA, Elwahy AHM, Abdelhamid IA, Abdelmoniem AM. Conjugate and regiochemical addition of aminoazoles to 2-(4-(2,2-dicyanovinyl)phenoxy)-N-arylacetamide affording fused pyrimidines linked to phenoxy-N-arylacetamide: antibacterial activity, molecular docking, and DNA binding studies. J Mol Struct. 2024;1307:137946. 10.1016/j.molstruc.2024.137946.

[72] Salem ME, El-Gabry YA, Abdelhamid IA, Elwahy AHM, Zaki MEA, Diab HM. Synthesis of Novel Diphenyl Ether-based Bis-Heterocycles as Novel Hybrid molecules via Michael and other Cyclocondensation reactions. ACS Omega. 2024;9:4073–84. 10.1021/acsomega.3c09081. 38284066 10.1021/acsomega.3c09081 PMC10809258

[73] El-Gabry YA, Salem ME, Ibrahim NS, Elwahy AHM, Abdelhamid IA, Diab HM. Novel diphenyl ether-heterocycles hybrids: synthesis via Hantzsch and Biginelli reactions, molecular docking simulation, and antimicrobial activities. J Mol Struct. 2024;1296:136857. 10.1016/j.molstruc.2023.136857.

[74] Ibrahim NS, Sayed HA, Sharaky M, Diab HM, Elwahy AHM, Abdelhamid IA. Synthesis, cytotoxicity, anti-inflammatory, anti-metastatic and anti-oxidant activities of novel chalcones incorporating 2-phenoxy-N-arylacetamide and thiophene moieties: induction of apoptosis in MCF7 and HEP2 cells. Naunyn Schmiedebergs Arch Pharmacol. 2024;397:10091–107. 10.1007/s00210-024-03255-9. 38980411 10.1007/s00210-024-03255-9 PMC11582173

[75] Abdelhamid IA, Darweesh AF, Elwahy AHM. Synthesis and characterization of poly(2,6-dimethyl-4-phenyl-1,4-dihydropyridinyl)arenes as novel multi-armed molecules. Tetrahedron Lett. 2015;56:7085–8.

[76] Abdelmoniem AM, Abdelhamid IA, Elwahy AHM, Abdelrahman MGM, Hassaneen HM, Teleb MAM. Synthesis of novel bis(spirocyclic-2-oxindole)-tethered 10b-azachrysene or 10a-azaphenanthrene systems via a Hantzsch-like reaction. Tetrahedron. 2024;162:134125. 10.1016/j.tet.2024.134125.

[77] Saleh FM, Hassaneen HM, Abdelhamid IA, Mohamed Teleb MA. Synthesis of novel spirocyclic 2-oxindole tethered to 2′-(3-(furan-2-yl)-1H-pyrazole-4-carbonyl)-hexahydropyrrolizine via 1,3-dipolar cycloaddition of the chalcone with azomethine ylide: reaction of pyrazolyl-enaminone towards some heteroaromatic amines. Tetrahedron Lett. 2024;137. 10.1016/j.tetlet.2024.154957. December 2023:154957.

[78] Abdelaal N, Ragheb MA, Hassaneen HM, Elzayat EM, Abdelhamid IA. Design, in silico studies and biological evaluation of novel chalcones tethered triazolo[3,4-a]isoquinoline as EGFR inhibitors targeting resistance in non-small cell lung cancer. Sci Rep. 2024;14:26647. https://www.nature.com/articles/s41598-024-76459-x. 39496648 10.1038/s41598-024-76459-x PMC11535068

[79] Elgamal AM, Abobakr E, Saad GR, Abdelhamid IA, Elsabee MZ, Hamed AA. Biologically active ionic chitosan Schiff base nanocomposites: synthesis, characterization and antimicrobial activity against Helicobacter pylori. Int J Biol Macromol. 2024;282:137321. 10.1016/j.ijbiomac.2024.137321. 39515719 10.1016/j.ijbiomac.2024.137321

[80] Barakat K, Ragheb MA, Soliman MH, Abdelmoniem AM, Abdelhamid IA. Novel thiazole– based cyanoacrylamide derivatives: DNA cleavage, DNA / BSA binding properties and their anticancer behaviour against colon and breast cancer cells. BMC Chem. 2024;18:183. 10.1186/s13065-024-01284-2. 39304938 10.1186/s13065-024-01284-2 PMC11414077

[81] Ibrahim NS, Mohamed MF, Elwahy AHM, Abdelhamid IA. Biological activities and Docking studies on Novel Bis 1,4-DHPS linked to Arene Core via Ether or Ester Linkage. Lett Drug Des Discov. 2018;15:1036–45.

[82] Abdelmoniem AM, Salaheldin TA, Abdelhamid IA, Elwahy AHM. New Bis(dihydropyridine-3,5-dicarbonitrile) derivatives: Green Synthesis and cytotoxic activity evaluation. J Heterocycl Chem. 2017;54:2670–7.

[83] Abdelmoniem AM, Ghozlan SAS, Abdelmoniem DM, Elwahy AHM, Abdelhamid IA. Facile One-pot, three-component synthesis of Novel Bis-heterocycles incorporating 5H-chromeno[2,3-b]pyridine-3-carbonitrile derivatives. J Heterocycl Chem. 2017;54:2844–9.

[84] Ghozlan SAS, Abdelmoniem AM, Abdelhamid IA. Chemistry of Azaenamines. Curr Org Chem. 2011;15:3098–119.

[85] Abdella AM, Abdelmoniem AM, Ibrahim NS, El-Hallouty SM, Abdelhamid IA, Elwahy AHM. Synthesis, cytotoxicity and molecular Docking Simulation of Novel bis-1,4-Dihydropyridines Linked to Aliphatic or Arene Core via Amide or Ester-Amide linkages. Mini Rev Med Chem. 2019;20:801–16. 10.2174/1389557519666190919160019. 10.2174/1389557519666190919160019 31538896

[86] Elwahy A, Shaaban M. Synthesis of pyrido- and pyrimido-fused heterocycles by multi-component reactions (part 3). Curr Org Synth. 2014;11:835–73.

[87] Salem ME, Darweesh AF, Farag AM, Elwahy AHM. 2-Bromo-1-(1H-pyrazol-4-yl)ethanone: versatile precursors for novel mono-, bis- and poly{6-(1H-pyrazol-4-yl)-[1,2,4]triazolo[3,4-b][1,3,4]thiadiazines}. Tetrahedron. 2016;72:712–9.

[88] Laohapaisan P, Chuangsoongnern P, Tummatorn J, Thongsornkleeb C, Ruchirawat S. Divergent synthesis of 3-Hydroxyfluorene and 4-Azafluorene derivatives from Ortho-Alkynylarylketones. J Org Chem. 2019;84:14451–60. 10.1021/ACS.JOC.9B01825/SUPPL_FILE/JO9B01825_SI_001.PDF. 31502842 10.1021/acs.joc.9b01825

[89] Wu YC, Duh CY, Wang SK, Chen KS, Yang TH. Two new natural azafluorene alkaloids and a cytotoxic aporphine alkaloid from polyalthia longifolia. J Nat Prod. 1990;53:1327–31. 10.1021/NP50071A028/ASSET/NP50071A028.FP.PNG_V03. 2292689 10.1021/np50071a028

[90] Prachayasittikul S, Manam P, Chinworrungsee M, Isarankura-Na-ayudhya C, Ruchirawat S, Prachayasittikul V. Bioactive azafluorenone alkaloids from Polyalthia debilis (pierre) finet & Gagnep. Molecules. 2009;14:4414–24. 10.3390/molecules14114414. 19924075 10.3390/molecules14114414 PMC6255371

[91] Venkateshan M, Muthu M, Suresh J, Ranjith Kumar R. Azafluorene derivatives as inhibitors of SARS CoV-2 RdRp: synthesis, physicochemical, quantum chemical, modeling and molecular docking analysis. J Mol Struct. 2020;1220:128741. 32834110 10.1016/j.molstruc.2020.128741 PMC7309803

[92] Hranjec M, Pavlović G, Karminski-Zamola G. Synthesis, crystal structure determination and antiproliferative activity of novel 2-amino-4-aryl-4,10-dihydro[1,3,5]triazino[1,2-a]benzimidazoles. J Mol Struct. 2012;1007:242–51.

