# Supplementary material for: Synthesis, and antibacterial activities of novel 1,3,4a,9-tetraza-4H-fluoren-2-amines incorporating phenoxy-N-arylacetamide, pyrazole, and 2-(4-(1-phenyl-1H-pyrazol-3-yl)phenoxy)-N-arylacetamide moieties
Source: BMC Chem. 2025 Mar 8;19(1):61. doi: 10.1186/s13065-025-01421-5 (PMC11890613; doi:10.1186/s13065-025-01421-5)
Supplement: Supplementary file 1 — Supplementary Material 1 [file 13065_2025_1421_MOESM1_ESM.docx]

**Supporting Information**

**Synthesis, antibacterial activities, and molecular docking of novel 1,3,4a,9-tetraza-4*H*-fluoren-2-amines incorporating phenoxy-*N*-arylacetamide, pyrazole, and 2-(4-(1-phenyl-1*H*-pyrazol-3-yl)phenoxy)-*N*-arylacetamide moieties**

Reham E. Abdelwahab^1^, Ahmed H. M. Elwahy^1*^, Nada S. Ibrahim,^2^ Amr M. Abdelmoniem^1^, Ismail A. Abdelhamid^1*^

^1^ Department of Chemistry, Faculty of Science, Cairo University, Giza 12613, Egypt.

E-mail:[aelwahy@cu.edu.eg](mailto:aelwahy@cu.edu.eg), [aelwahy@hotmail.com](mailto:aelwahy@hotmail.com), [ismail_shafy@yahoo.com](mailto:ismail_shafy@yahoo.com), [ismail_shafy@cu.edu.eg](mailto:ismail_shafy@cu.edu.eg)

^2^Department of Chemistry (Biochemistry Division), Faculty of Science, Cairo University, Giza, 12613, Egypt

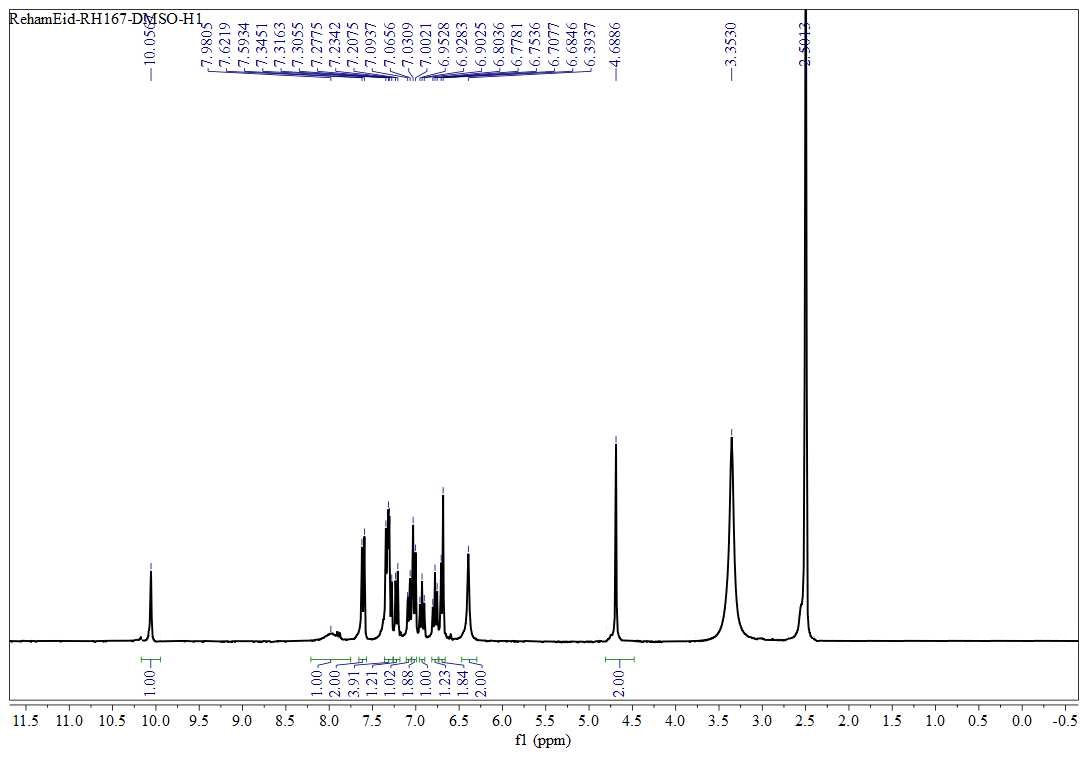


**Figure 1**. ^1^H NMR spectrum of compound **5a**

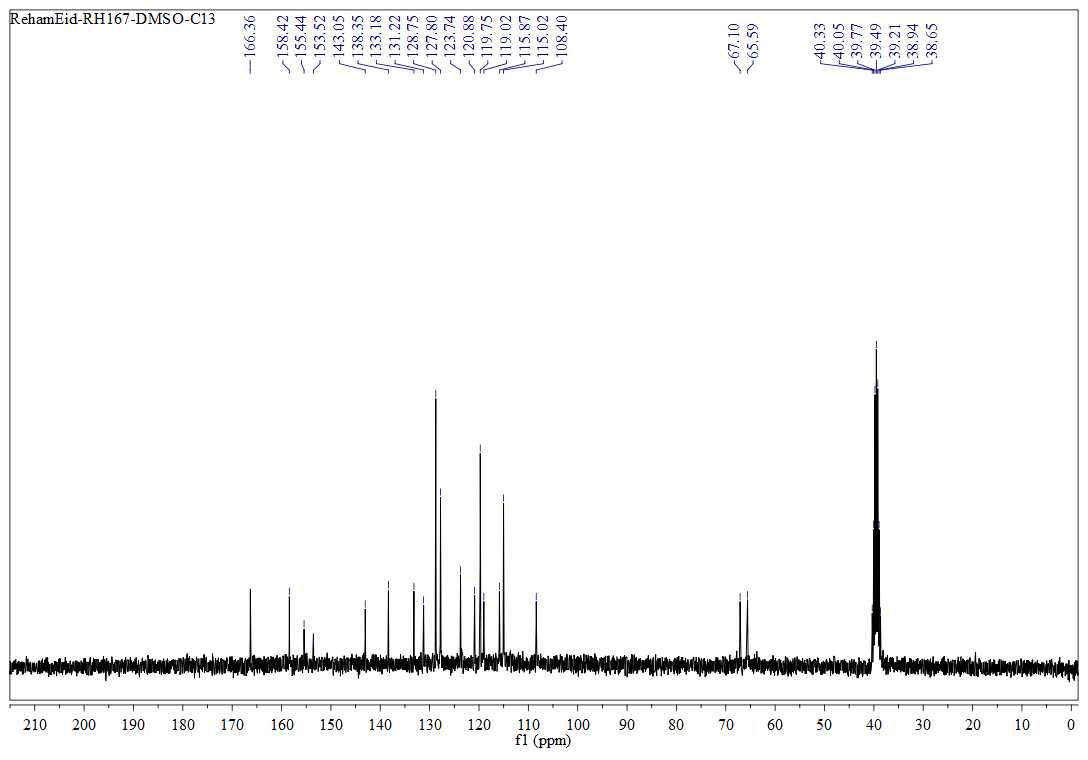


**Figure 2**. ^13^C NMR spectrum of compound **5a**

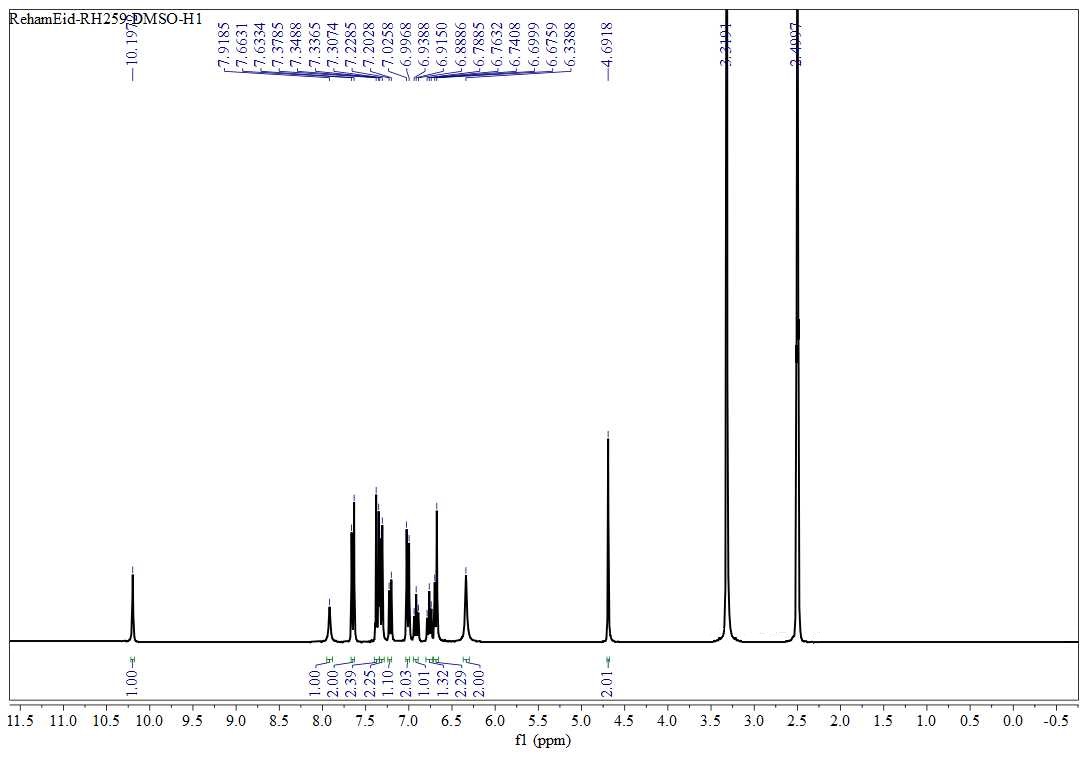


**Figure 3**. ^1^H NMR spectrum of compound **5b**

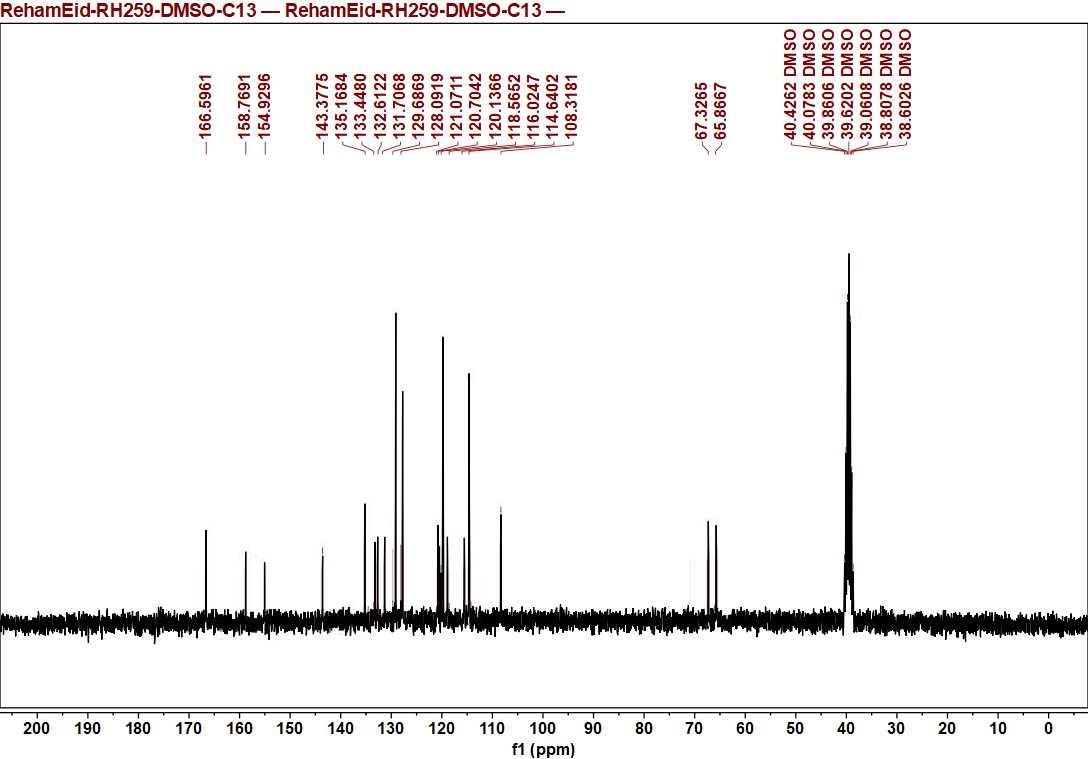


**Figure 4**. ^13^C NMR spectrum of compound **5b**

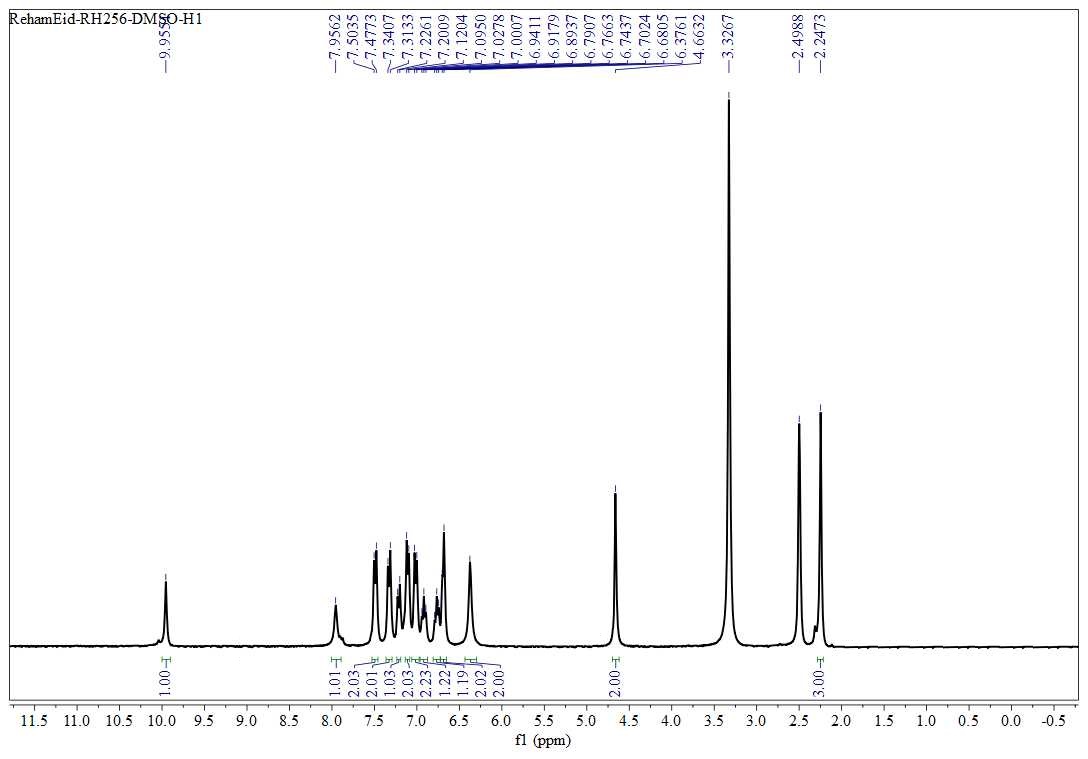


**Figure 5**. ^1^H NMR spectrum of compound **5c**

**
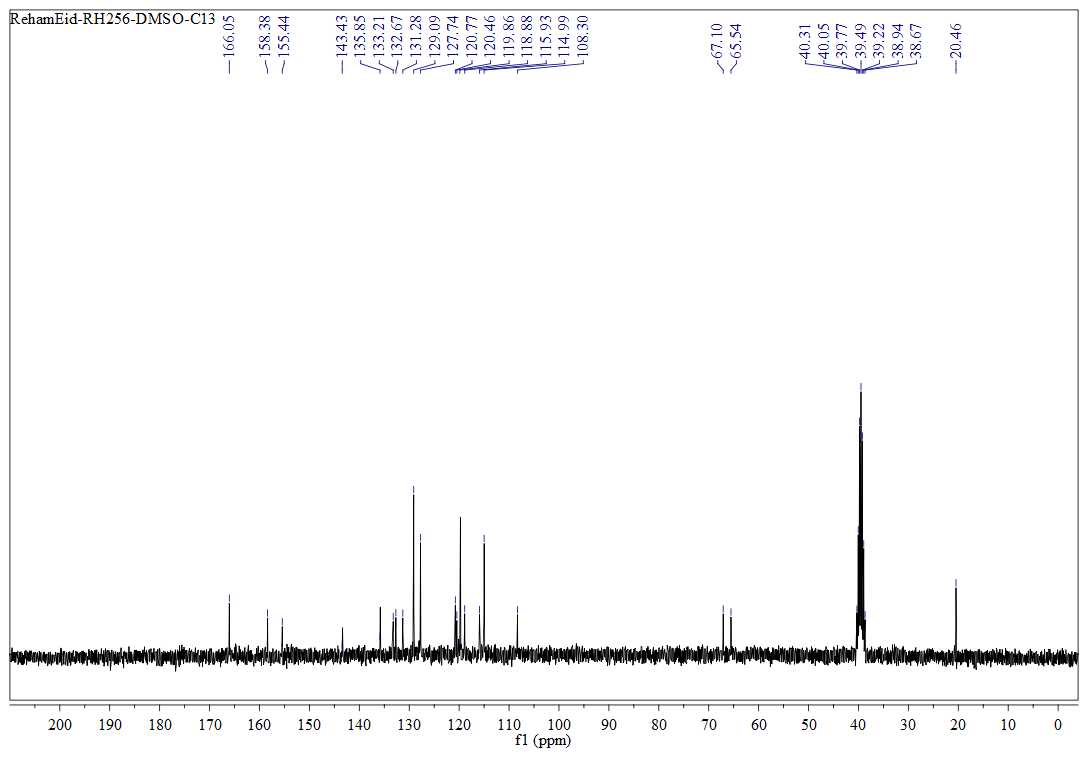
**

**Figure 6**. ^13^C NMR spectrum of compound **5c**

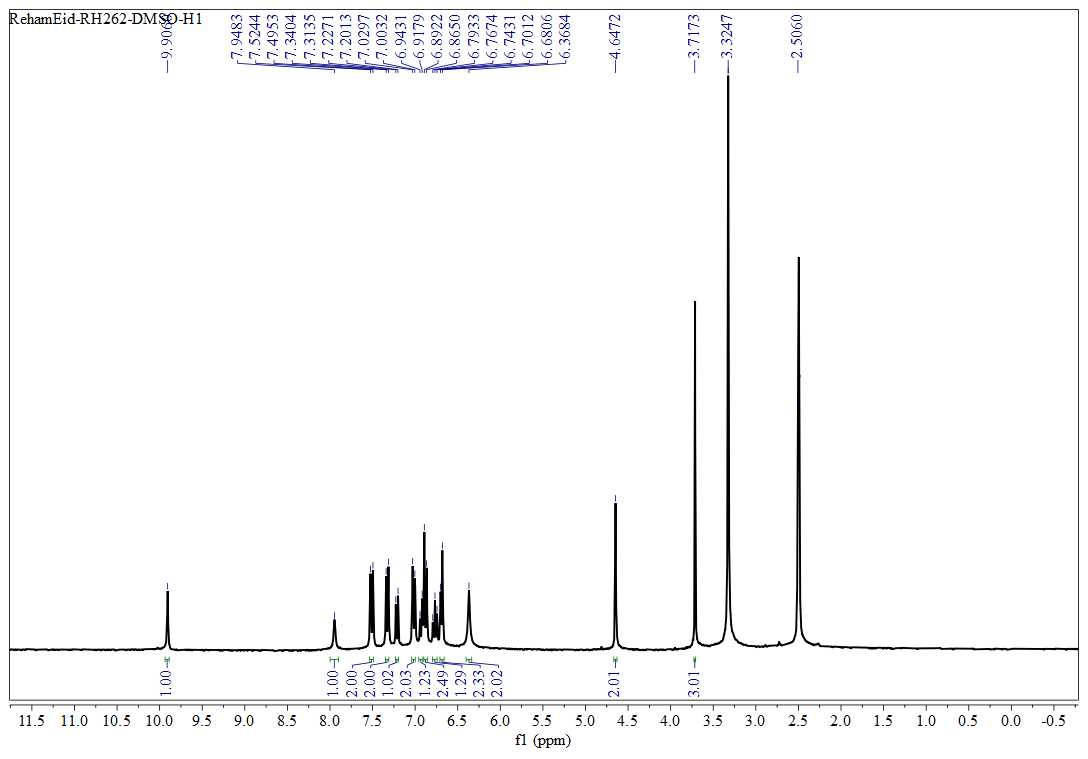


**Figure 7**. ^1^H NMR spectrum of compound **5d**

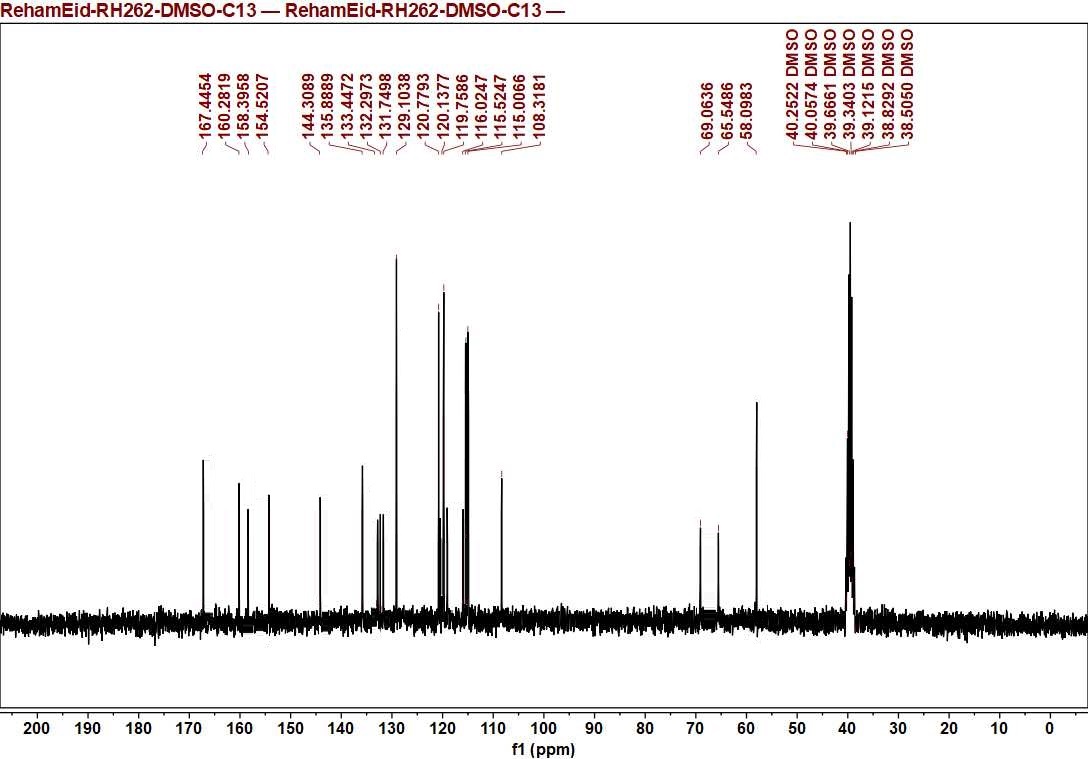
 **Figure 8**. ^13^C NMR spectrum of compound **5d**

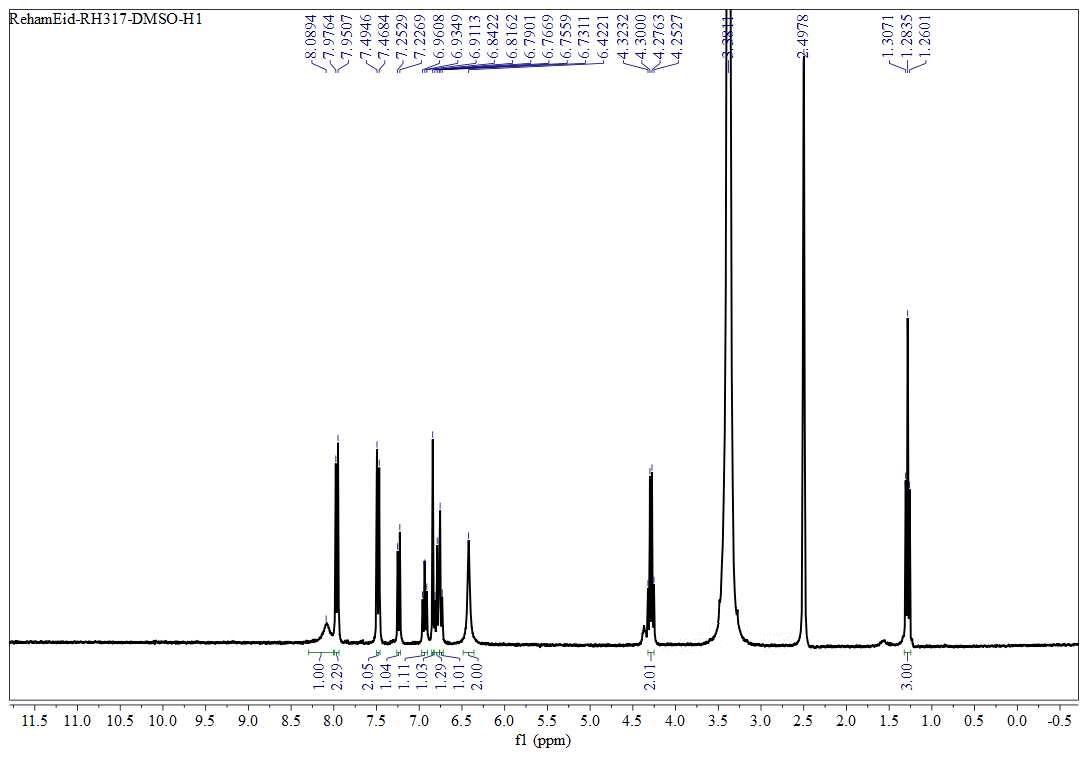


**Figure 9**. ^1^H NMR spectrum of compound **8**

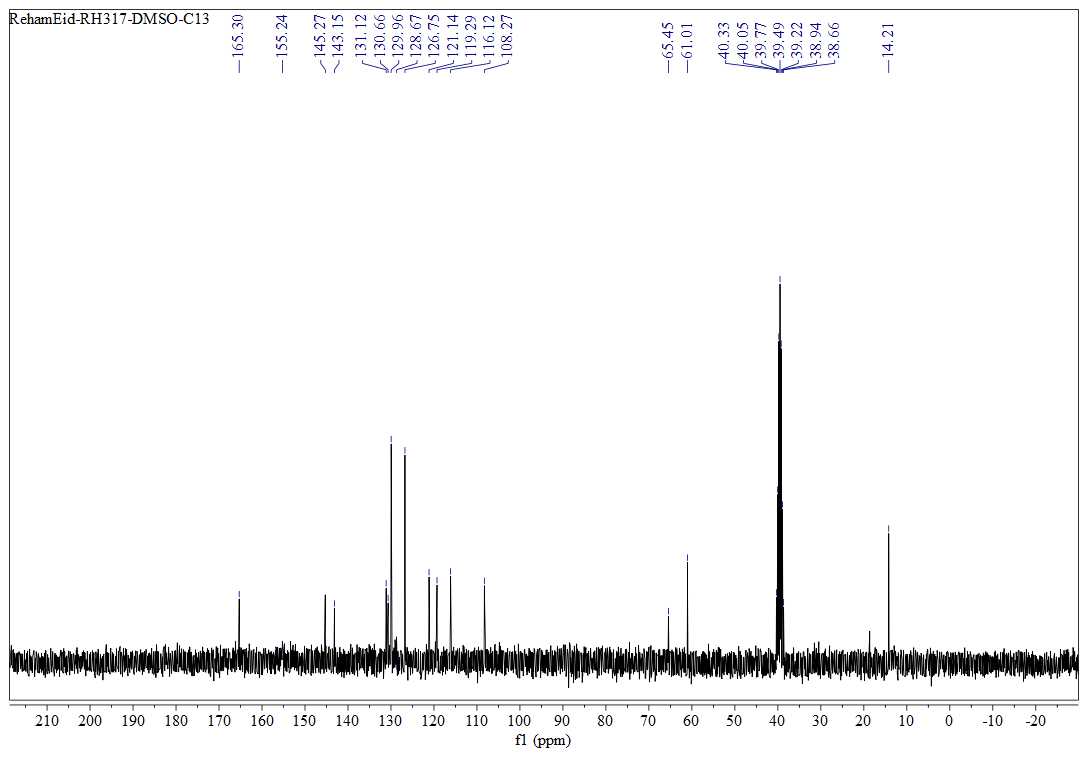


**Figure 10**. ^13^C NMR spectrum of compound **8**

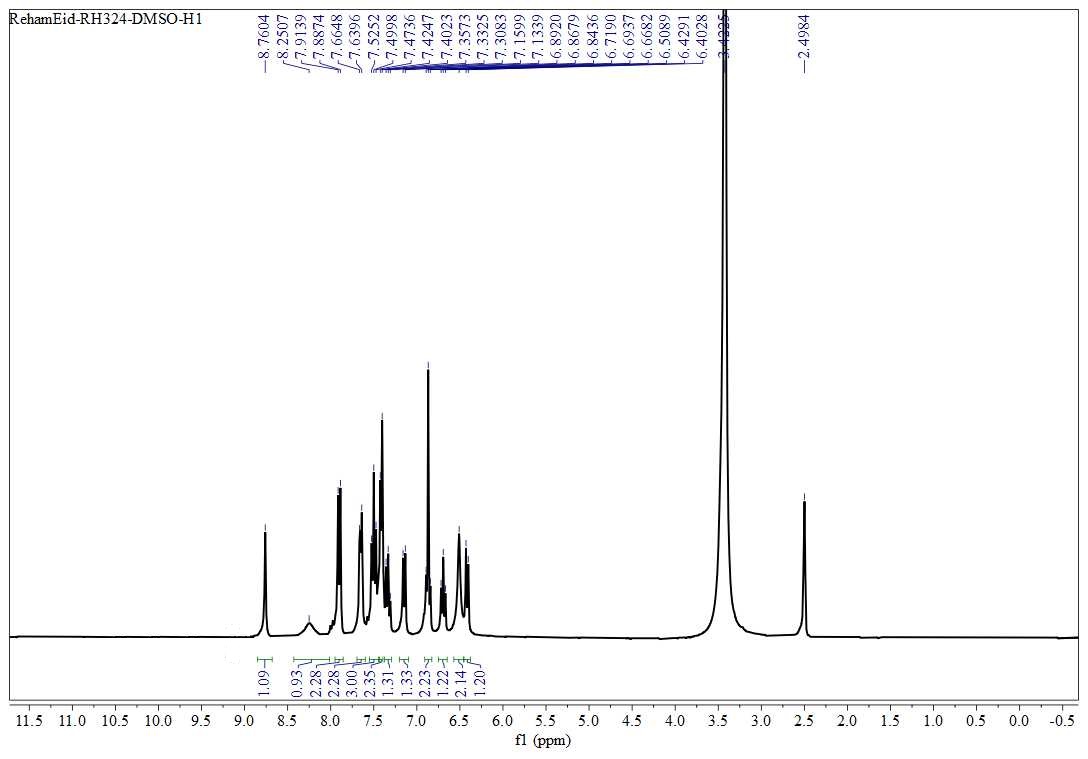


**Figure 11**. ^1^H NMR spectrum of compound **12a**

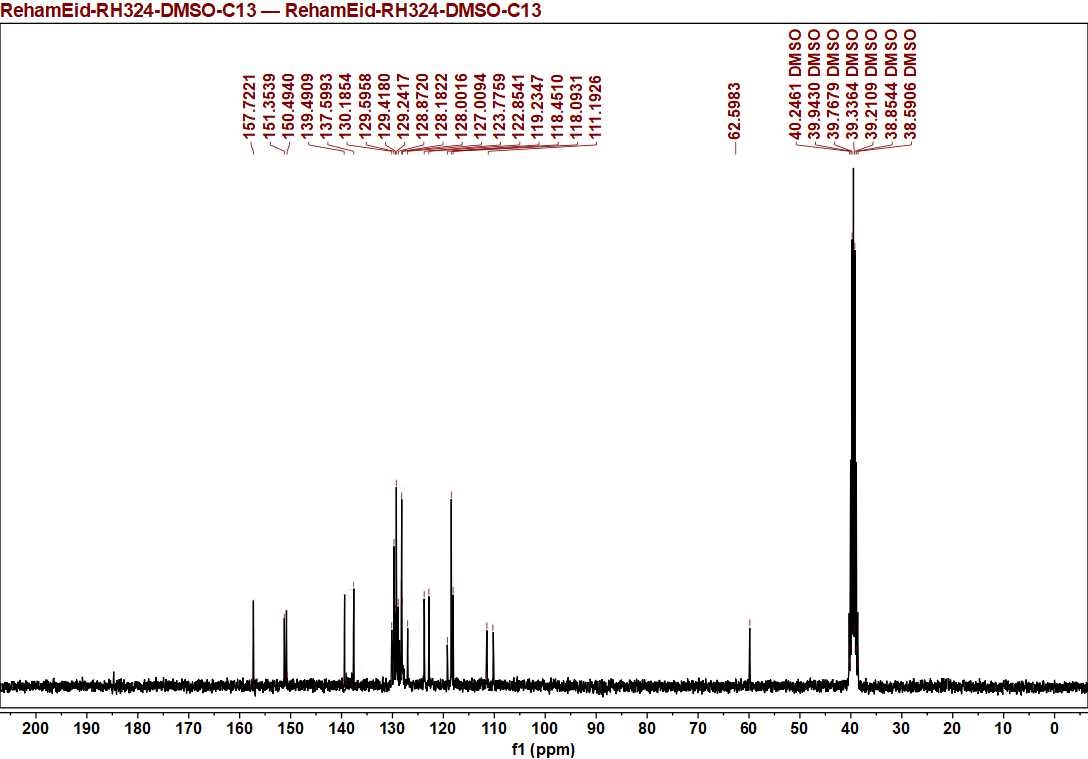


**Figure 12**. ^1^H NMR spectrum of compound **12a**

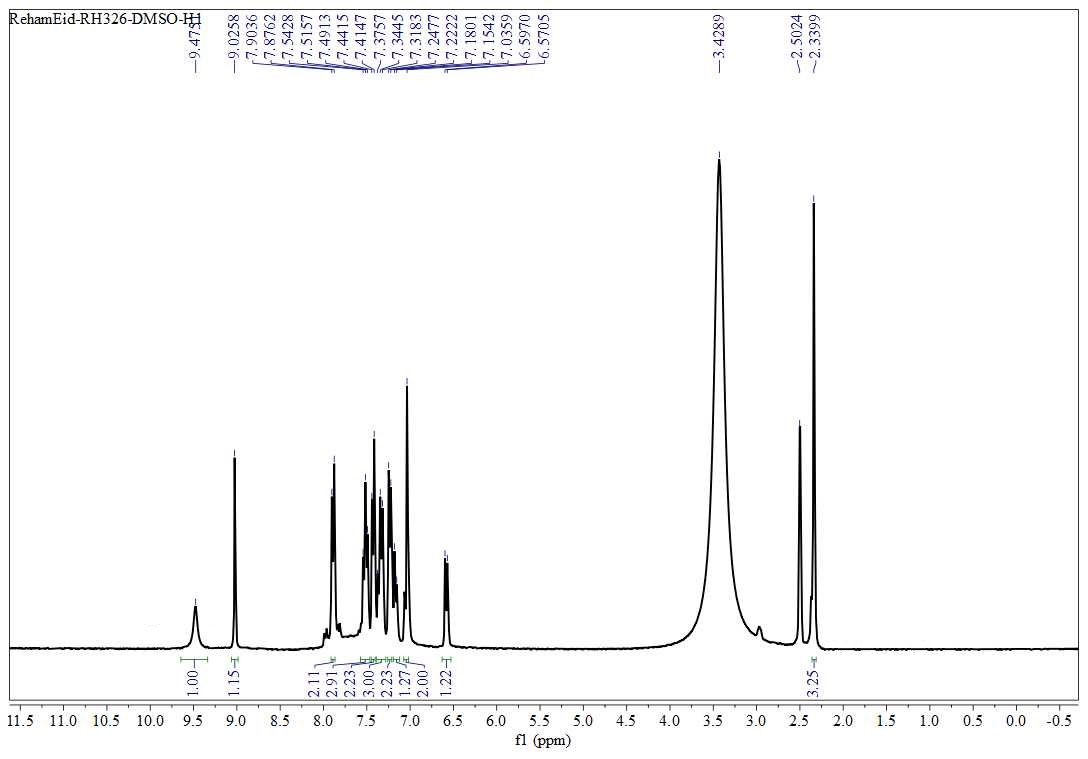


**Figure 13**. ^1^H NMR spectrum of compound **12b**

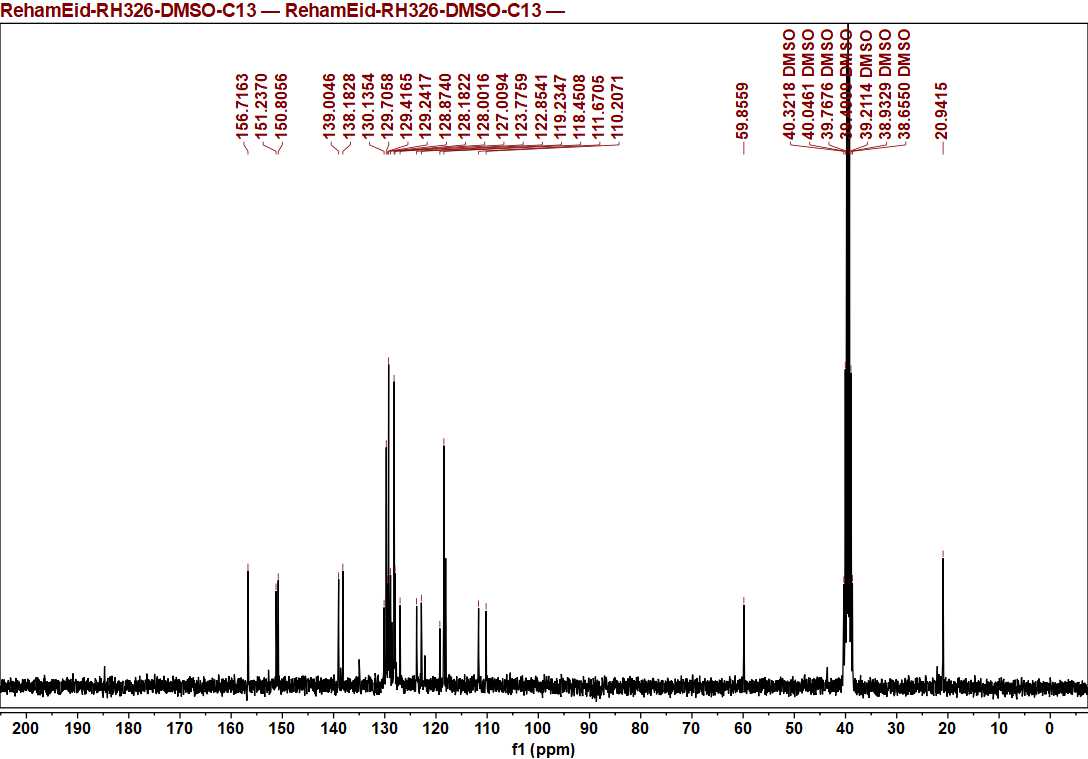


**Figure 14**. ^13^C NMR spectrum of compound **12b**

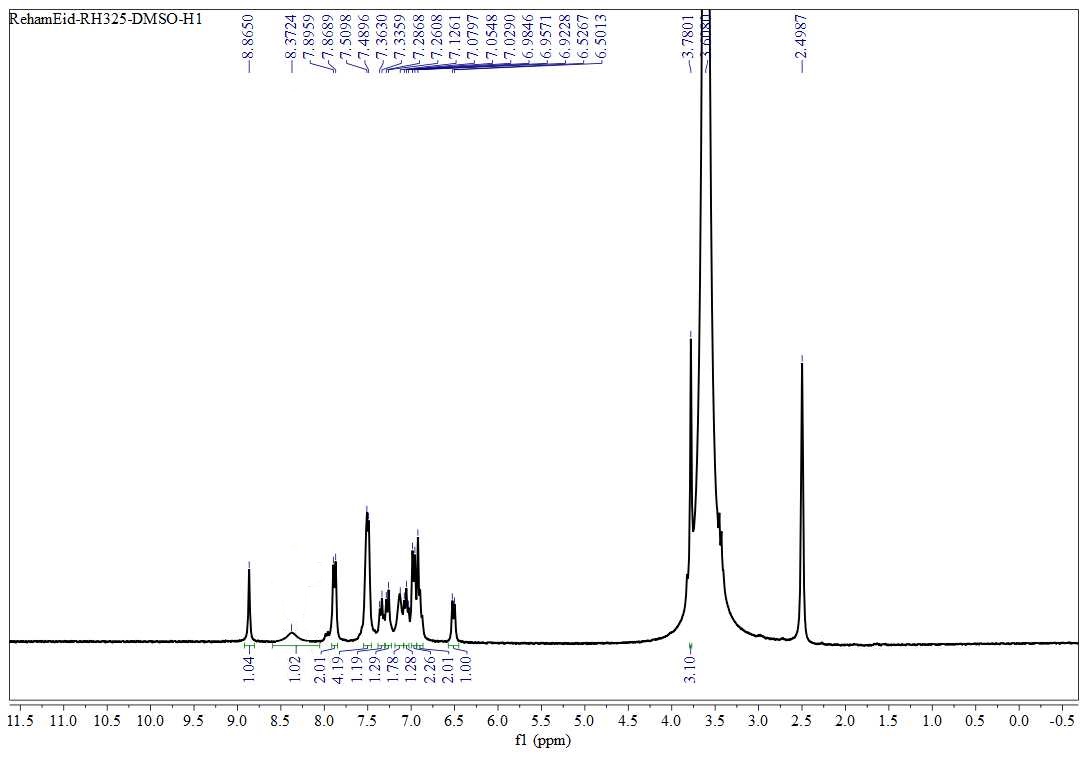


**Figure 15**. ^1^H NMR spectrum of compound **12c**

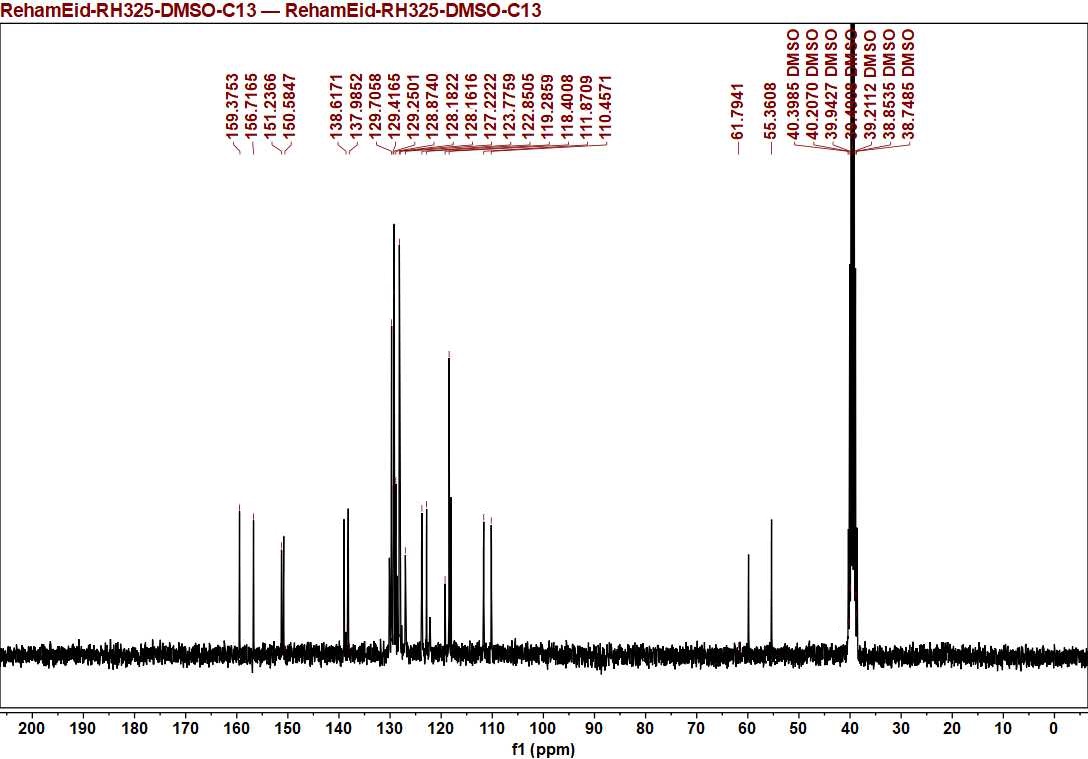


**Figure 16**. ^1^H NMR spectrum of compound **12c**

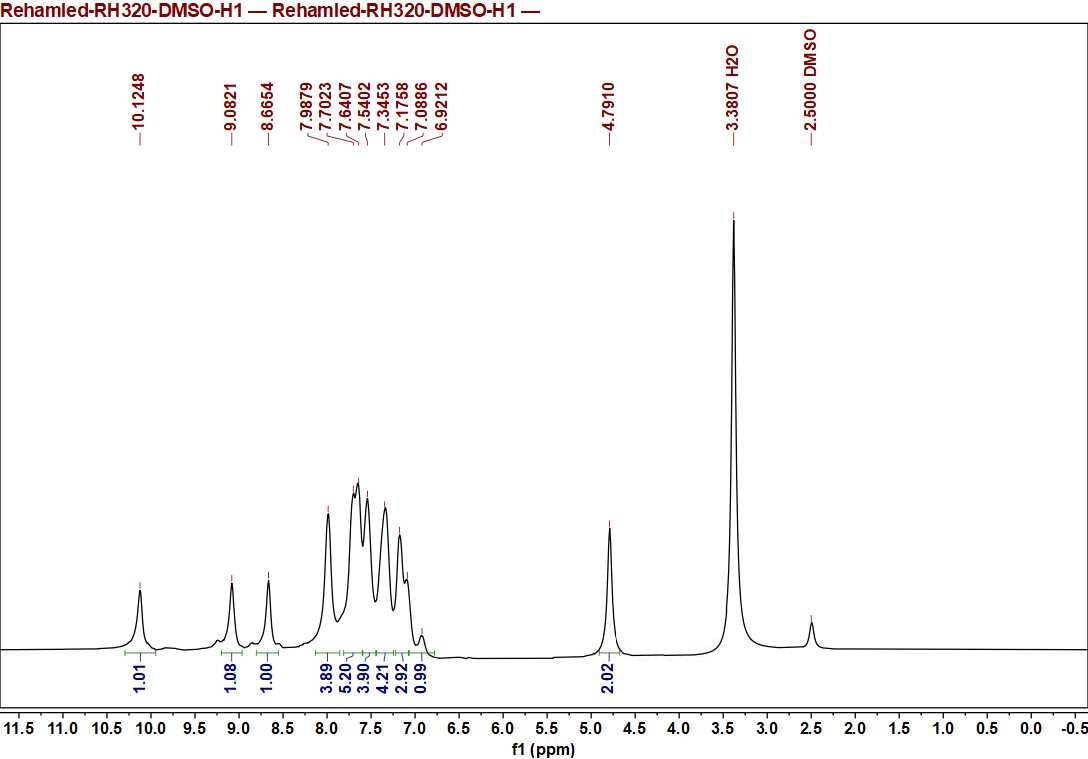


**Figure 17**. ^1^H NMR spectrum of compound **14a**

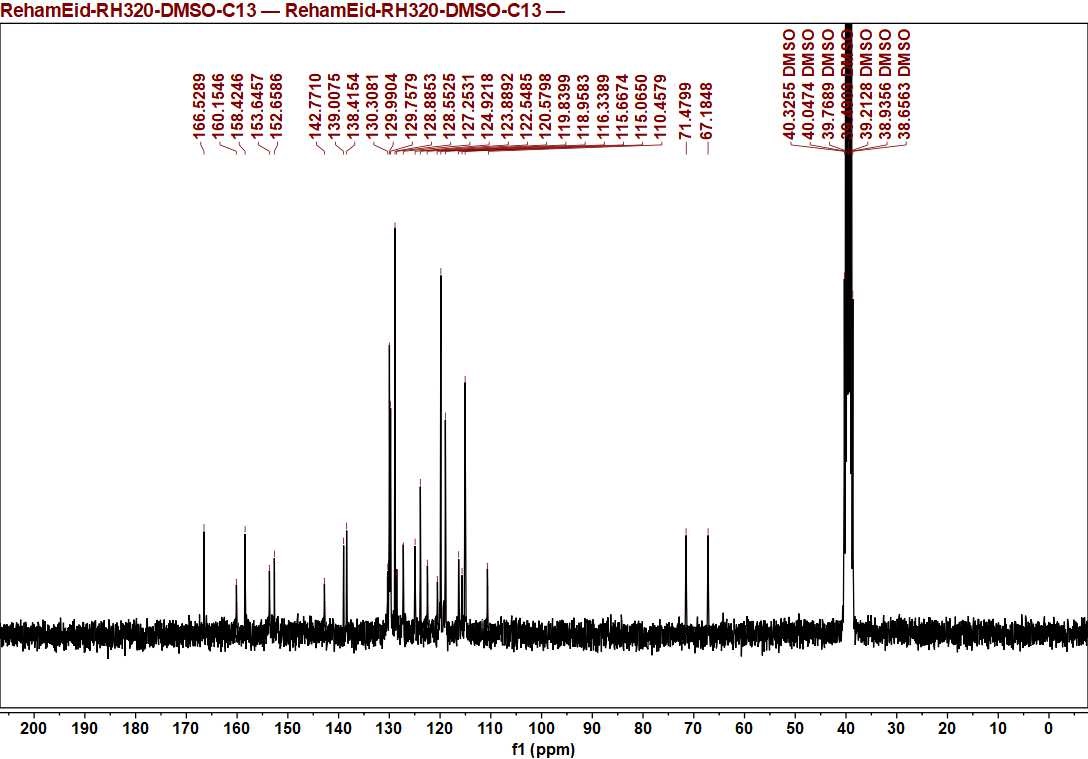


**Figure 18**. ^13^C NMR spectrum of compound **14a**

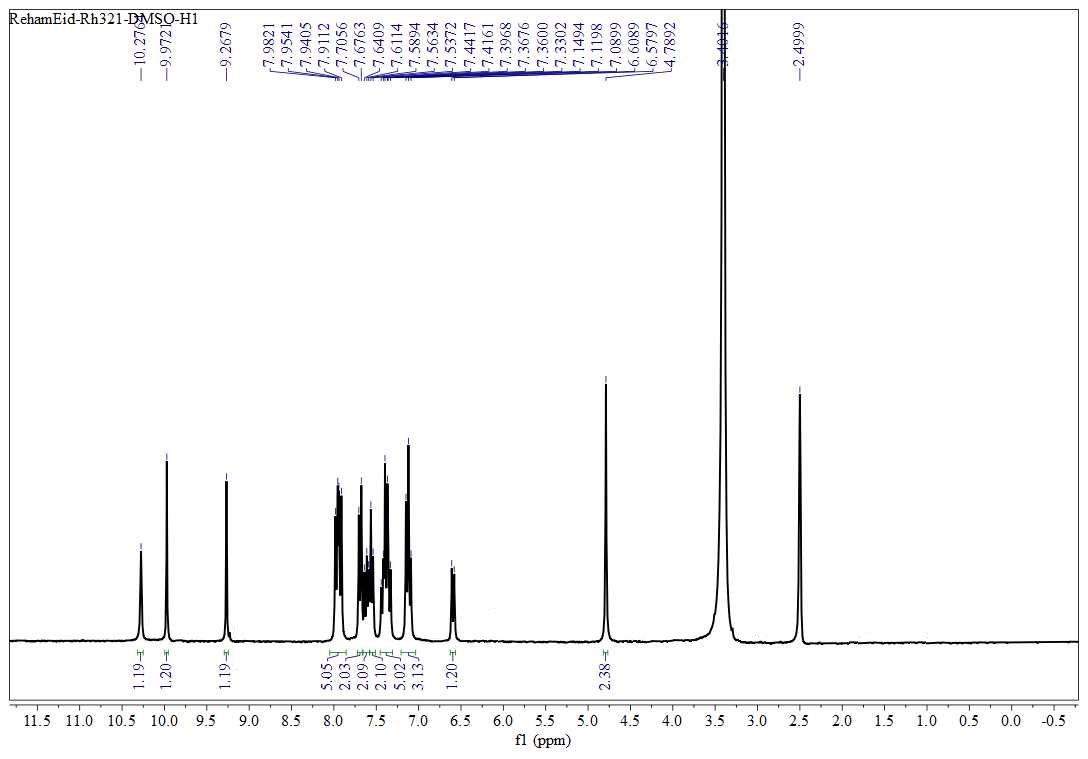


**Figure 19**. ^1^H NMR spectrum of compound **14b**

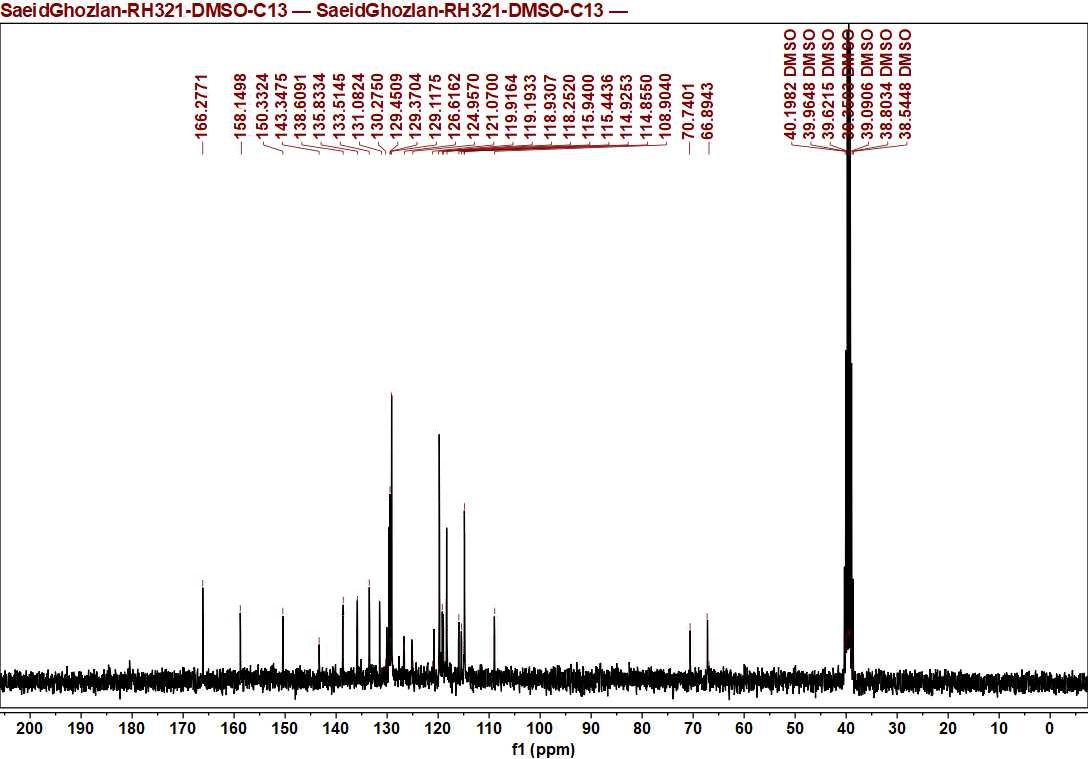


**Figure 20**. ^13^C NMR spectrum of compound **14b**

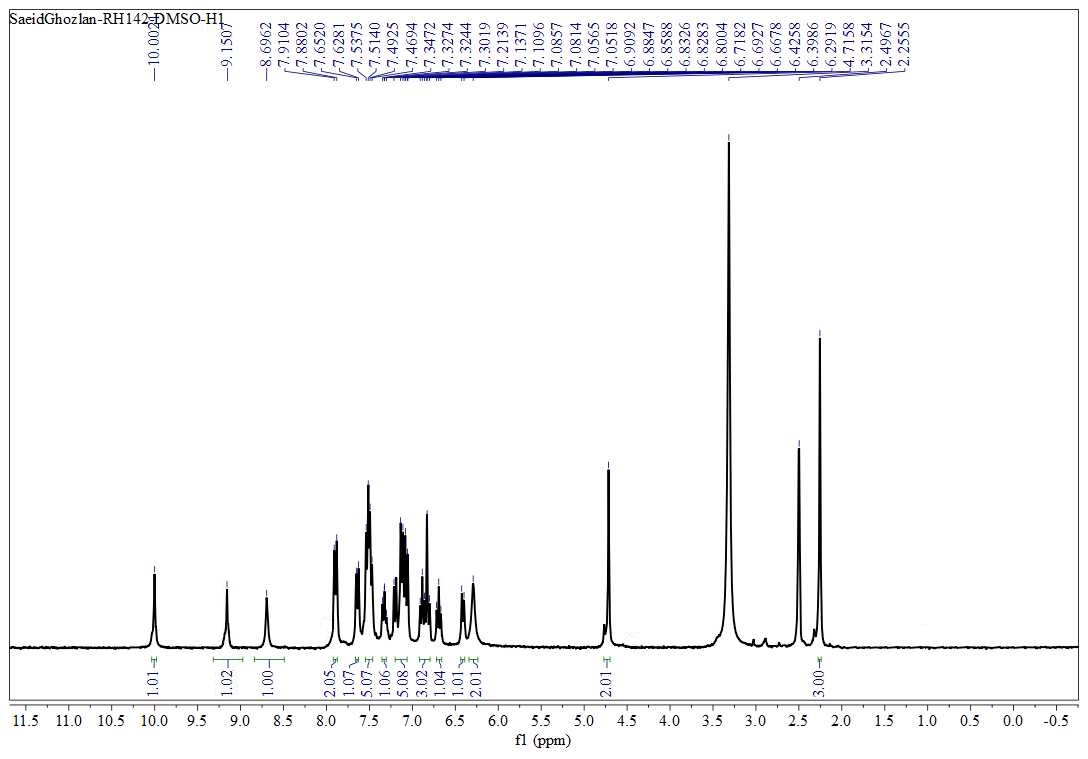


**Figure 21**. ^1^H NMR spectrum of compound **14c**

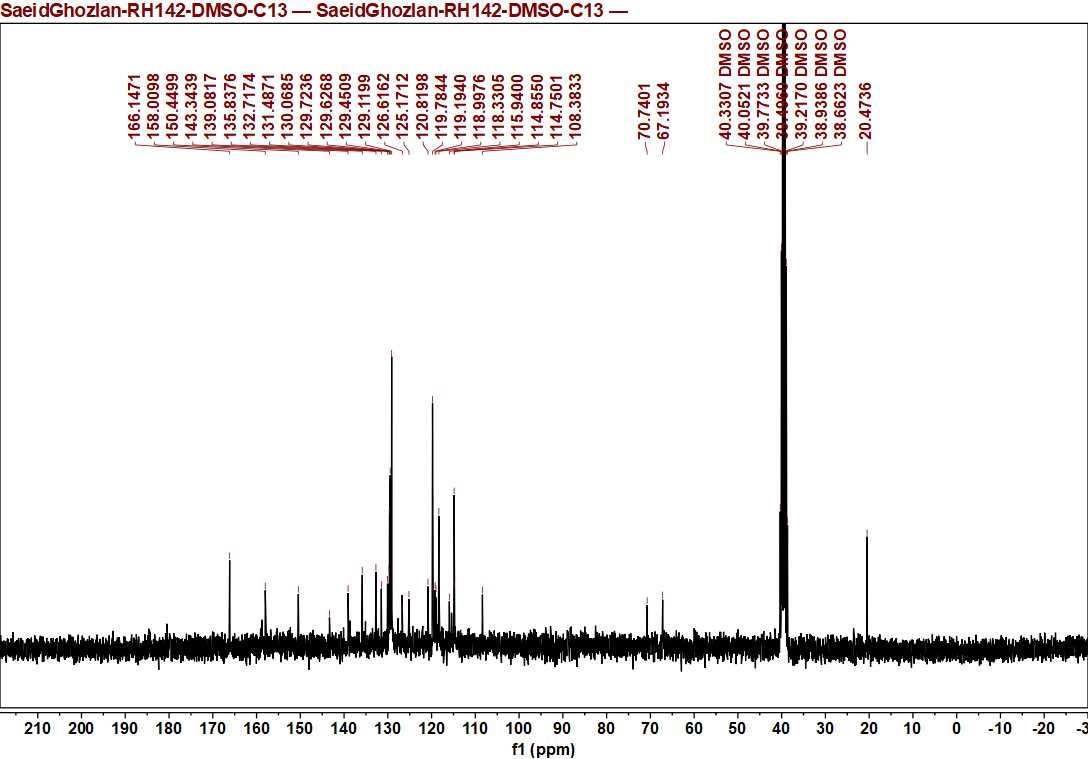


**Figure 22**. ^13^C NMR spectrum of compound **14c**

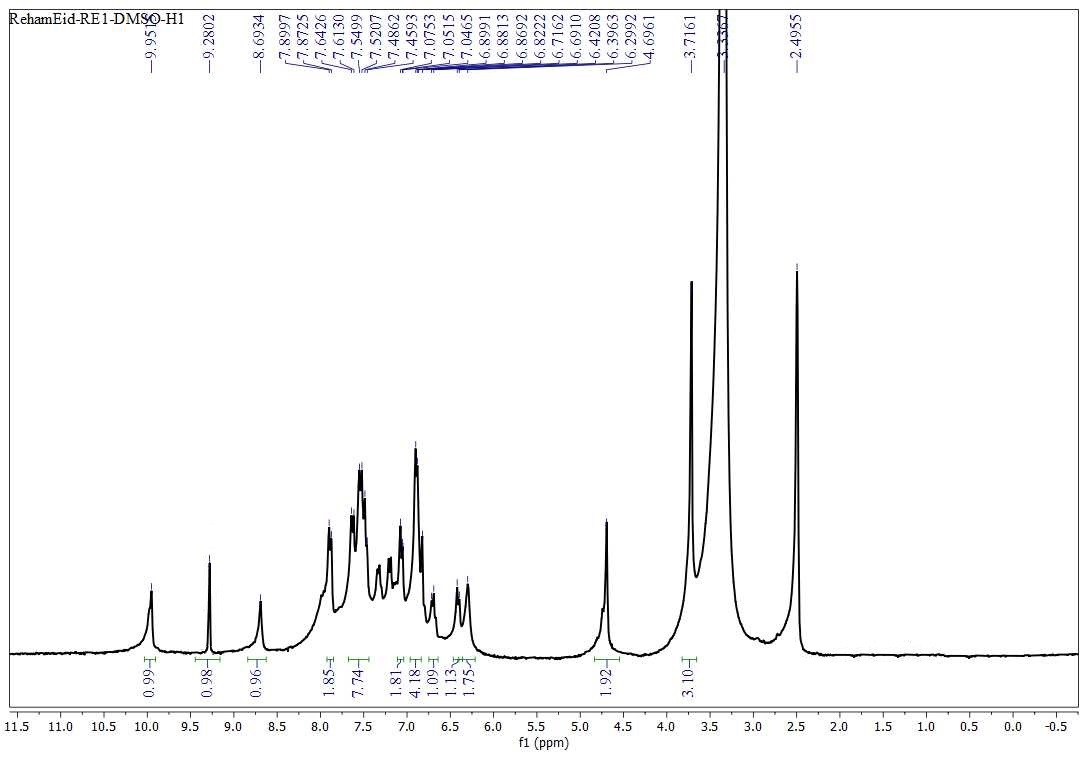


**Figure 23**. ^1^H NMR spectrum of compound **14d**
